# Integrative network-based analysis of mRNA and microRNA expression in 1,25-dihydroxyvitamin D_3_-treated cancer cells

**DOI:** 10.1007/s12263-015-0484-0

**Published:** 2015-08-15

**Authors:** Martina Kutmon, Susan L. Coort, Kim de Nooijer, Claire Lemmens, Chris T. Evelo

**Affiliations:** Department of Bioinformatics - BiGCaT, NUTRIM School for Nutrition, Toxicology and Metabolism, Maastricht University, Maastricht, The Netherlands; Maastricht Centre for Systems Biology (MaCSBio), Maastricht University, Maastricht, The Netherlands

**Keywords:** Pathway analysis, Network analysis, Vitamin D, Prostate cancer, Systems nutrition

## Abstract

Nutritional systems biology is an evolving research field aimed at understanding nutritional processes at a systems level. It is known that the development of cancer can be influenced by the nutritional status, and the link between vitamin D status and different cancer types is widely investigated. In this study, we performed an integrative network-based analysis using a publicly available data set studying the role of 1,25-dihydroxyvitamin D_3_ (1,25(OH)_2_D_3_) in prostate cancer cells on mRNA and microRNA level. Pathway analysis revealed 15 significantly altered pathways: eight more general mostly cell cycle-related pathways and seven cancer-specific pathways. The changes in the G1-to-S cell cycle pathway showed that 1,25(OH)_2_D_3_ down-regulates the genes influencing the G1-to-S phase transition. Moreover, after 1,25(OH)_2_D_3_ treatment the gene expression in several cancer-related processes was down-regulated. The more general pathways were merged into one network and then extended with known protein–protein and transcription factor–gene interactions. Network algorithms were used to (1) identify active network modules and (2) integrate microRNA regulation in the network. Adding microRNA regulation to the network enabled the identification of gene targets of significantly expressed microRNAs after 1,25(OH)_2_D_3_ treatment. Six of the nine differentially expressed microRNAs target genes in the extended network, including *CLSPN*, an important checkpoint regulator in the cell cycle that was down-regulated, and *FZD5*, a receptor for Wnt proteins that was up-regulated. The extendable network-based tools PathVisio and Cytoscape enable straightforward, in-depth and integrative analysis of mRNA and microRNA expression data in 1,25(OH)_2_D_3_-treated cancer cells.

**Electronic supplementary material:**

The online version of this article (doi:10.1007/s12263-015-0484-0) contains supplementary material, which is available to authorized users.

## Introduction

Nutritional systems biology is an evolving research field aimed at understanding nutritional processes at a systems level. Integrating the effects of nutritional compounds at the gene expression level with information about the regulatory level can shed a new light on their action mechanism. Promising regulatory molecules in this respect are small non-coding RNAs, like microRNAs, which regulate gene expression post-transcriptionally. Nowadays high-throughput technologies enable the measurements of mRNA and microRNA expression on a large scale. Pathway- and network-based approaches are meaningful for the integrated analysis of these two types of omics data. The present study demonstrates how pathway analysis can be combined with network analysis to perform an integrated analysis of transcriptomics and microRNA-omics data. The integrative analysis uses two widely adopted open source tools for pathway and network analysis: PathVisio (Kutmon et al. 2015) and Cytoscape (Shannon et al. 2003).

### System nutrition

Nutritional status is known to influence cancer development (Andreoli et al. 2011; McMillan 2009). The link between the vitamin D status and different types of cancer is widely investigated (Hatse et al. 2012; Shui and Giovannucci 2014). The present study explores the role of the biologically active form of vitamin D3, 1,25-dihydroxyvitamin D_3_ (1,25(OH)_2_D_3_), in prostate cancer. Several studies suggest that adequate vitamin D levels have a protective effect with respect to the development of prostate cancer, and it is believed that vitamin D has chemopreventive properties. Recently, a study by Wang et al. (2011) investigated the 1,25(OH)_2_D_3_-mediated intracellular pathways by measuring global gene expression in LNCaP prostate cancer cells. In addition, they examined the expression of microRNAs in these cells upon treatment with 1,25(OH)_2_D_3_. Wang et al. (2011) clearly showed that 1,25(OH)_2_D_3_ can modulate gene and microRNA profiles in LCNaP cells and affects processes involved in cell cycle arrest, calcium ion homoeostasis and phosphoinositide-mediated signalling. In the present study, we will combine pathway- and network-based methods to decipher the regulatory action of 1,25(OH)_2_D_3_ in prostate cancer cells on mRNA and microRNA level.

## Materials and methods

### Transcriptomics and microRNA data sets

In the Gene Expression Omnibus (GEO, http://ncbi.nlm.nih.gov/geo/, Barrett and Edgar 2006), we found two studies in which global gene expression was measured in 1,25(OH)_2_D_3_-treated prostate cells, accession numbers: GSE17461 and GSE15947. After a thorough analysis, we selected the study by Wang et al. (2011) because they used prostate cancer cells (LNCaP cells), whereas Kovalenko et al. (2010) used non-tumourigenic prostate cells (RWPE1 cells). Moreover, only in the LNCaP cells the microRNA expression was measured in addition to the mRNA expression, thereby adding the needed regulatory level.

The mRNA and microRNA expression measured in the study by Wang et al. (2011) was used in this analysis. These data are published and publicly available in GEO (accession numbers GSE17461 and GSE23814). LNCaP human prostate cancer cells were plated at a density of 1 × 10^6^ cells per 150 cm^2^ for 48 h prior to treatment with 100 nM 1,25(OH)_2_D_3_; see Wang et al. (2011) for further details. Total RNA was isolated from the LNCaP cells using standard protocols. Gene expression was measured with Nimblegen-HG18-4plex whole-genome microarrays in 1,25(OH)_2_D_3_-treated (*n* = 3) and non-treated (control, *n* = 3) LNCaP cells. Agilent human microRNA v3 microarrays were used to measure microRNA expression in 1,25(OH)_2_D_3_-treated (*n* = 4) and non-treated (control, *n* = 4) cells.

Further pre-processing and quality control were performed by Wang et al. (2011) as previously described. GeneSpring GX10 software was used to check the data quality of the raw data and to normalize the data with RNA. They filtered the obtained gene list to remove probes that showed a low signal value (i.e. bottom 20th percentile) over all samples. Thereafter they used one-way ANOVA (*p* < 0.05) to find significantly changed genes when comparing 1,25(OH)_2_D_3_-treated cells versus non-treated cells. Within each test, correction for multiple testing was performed. A fold change (FC) cut-off at 1.5-fold was used to obtain a final list of differentially expressed genes.

Also the raw microRNA data were pre-processed in GeneSpring GX10. In the microRNA list, probes with a low signal (i.e. bottom 20th percentile) were excluded in all groups. In addition, only probes which were present in at least three biological replicates and in all groups were taken into account. MicroRNAs with fold changes greater than 2.0 were considered significant when *p* < 0.05 using one-way ANOVA with correction for multiple testing. In the next analysis steps, the statistically analysed data of both gene expression and microRNA expression generated by Wang et al. (2011) were used.

### Pathway analysis

Pathway analysis was performed using PathVisio (version 3.1.3), a commonly used tool to create, visualize and analyse biological pathways (Kutmon et al. 2015). The human pathway collection from WikiPathways [Kelder et al. (2012), curated collection with 276 pathways download on 29 January 2015] was used to perform an over-representation analysis with the transcriptomics data set.

The pathways were then ranked based on a standardized difference score (*Z* score). A pathway was considered altered when (1) *Z* score >1.96, (2) permutation *p* value <0.05 and (3) minimum number of differentially expressed genes (|FC| > 1.5, *p* value <0.05) in the pathway is five.

### Network analysis

#### Network of interconnected pathways

Cytoscape is a widely adopted network visualization and analysis tool (Shannon et al. 2003). In this study, the WikiPathways app for Cytoscape (Kutmon et al. 2014) was used to load the altered pathways as networks and then merge them into one large network using Cytoscape’s merge function. An identifier mapping step was performed using the BridgeDb app for Cytoscape to unify the identifiers in the selected pathways (Gao et al. 2014). Thus, unified pathway elements, i.e. gene products, metabolites or pathway nodes, that are present in two or more pathways are linking the pathways to each other in the network.

#### Network extension

The network of interconnected pathways was extended with known protein–protein and transcription factor–target interactions (first neighbours) between the genes in the pathways and all other differentially expressed genes. Protein–protein interactions were obtained from the STRING database (Franceschini et al. 2013, medium confidence level, score >0.4), and the transcription factor–target interactions were extracted from the ENCODE project (Gerstein et al. 2012). The created network will be addressed as the vitamin D-extended network.

#### Active network modules

Active network modules are small, connected subnetworks that contain genes that show significant changes in expression. The jActiveModules app in Cytoscape was used to identify active modules in the vitamin D-extended network (Ideker et al. 2002). We selected the highest scoring active module and used the ClueGO app (version 2.1.5) for Cytoscape to perform a functional analysis (Bindea et al. 2009). It performs an enrichment analysis of the genes in the active module to find relevant Gene Ontology (GO) classes. A ClueGO network was created with kappa statistics, which reflects the relationships between the GO classes, based on the similarity of their associated genes.

#### Vitamin D-microRNA network

Using the CyTargetLinker app in Cytoscape (Kutmon et al. 2013), microRNA–target gene interactions from miRTarBase (Hsu et al. 2014, version 4.5) and TargetScan (Grimson et al. 2007, version 6.2) were added to the network. A subnetwork of differentially expressed microRNAs in the 1,25(OH)_2_D_3_-treated cells with their target genes was created. In this step, the microRNA and mRNA expression levels were integrated and visualized together in the subnetwork.

#### Vitamin D receptor target analysis

In a literature search in NCBI PubMed (www.ncbi.nlm.nih.gov/pubmed), we manually extracted 178 human vitamin D receptor (VDR) target genes from 25 different articles and books. First, the studies of interest were collected based on the following search terms: “VDR target gene”, “vitamin D receptor”, “VDR” or “gene regulation vitamin D”. These search terms were also used to find relevant information in (online) books at the Maastricht University Library. Second, relevant studies were selected when the title and/or abstract included information on VDR target genes. Third, the methods used to determine the VDR target genes were manually verified and included ChIP sequencing, RNA sequencing and microarrays. Finally, all human VDR target genes from the selected studies were included in the analysis. Some of the target genes were reported in up to seven different articles, others only in one; see Supplementary Material 1. In the interpretation of the network of interconnected pathways and the extended network, the presence and location of the VDR target genes were investigated.

## Results and discussion

**Fig. 1:**
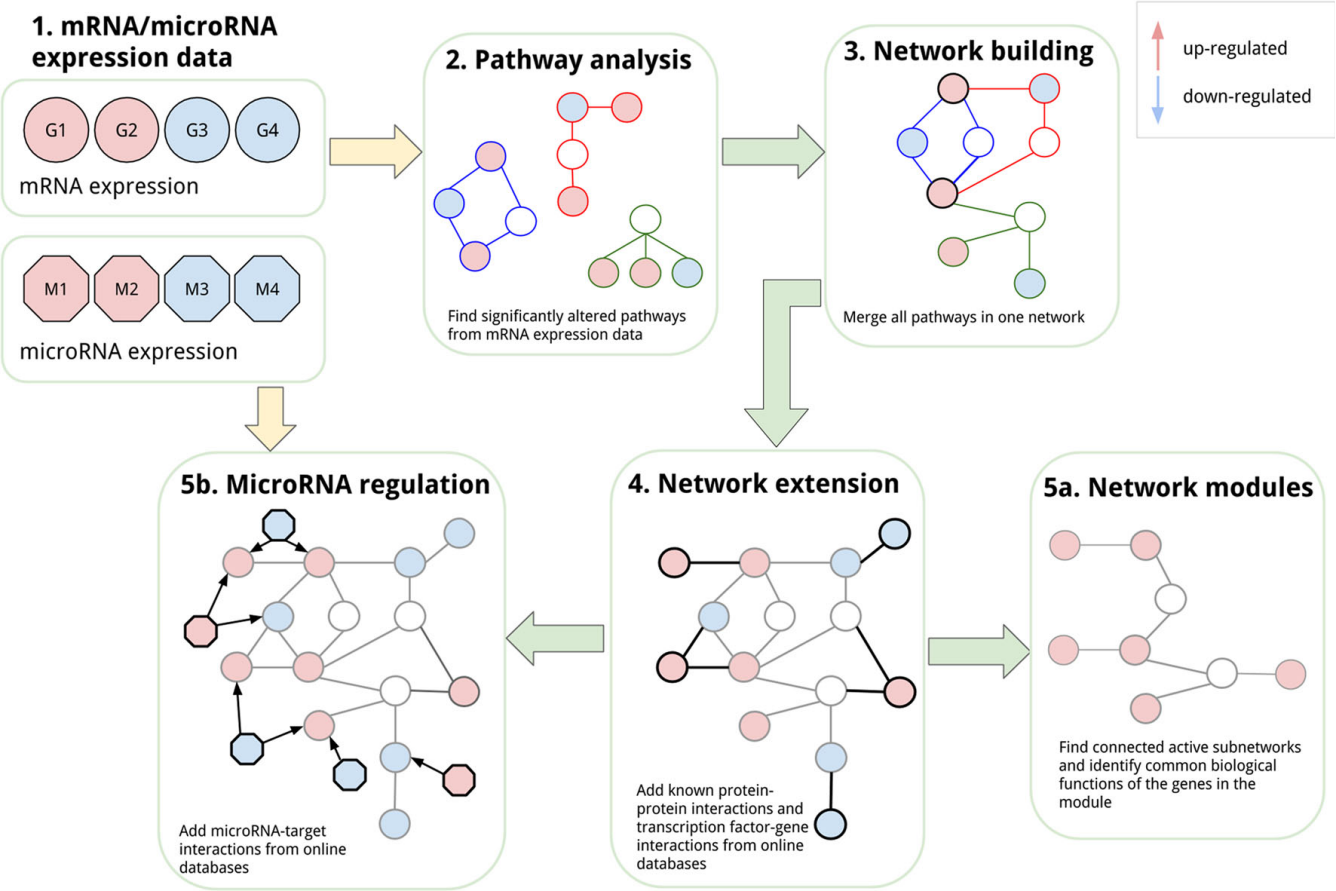
Integrative network-based analysis. This overview figure highlights the different steps in the integrative network-based analysis used in this study. The goal is to integrate different omics data sets like mRNA and microRNA expression data (*1*). First, the mRNA expression data were analysed using biological pathways and significantly altered pathways were identified (*2*). The selected pathways were then merged into one network (*3*). In the next step, the network was extended with protein–protein and transcription factor–gene interactions with other differentially expressed genes that are not present in the pathways (*4*). The extended network was used to first identify active modules (*5a*), and then, it was extended with microRNA regulation, which allowed the integration of microRNA expression data (*5b*)

In this section, the six steps of our analysis will be presented. The basic principles are shown in Fig. 1. The goal is the integrative analysis of transcriptomics and microRNA expression data using pathway- and network-based approaches. Starting with pathway analysis of the transcriptomics data set, it was possible to identify a set of altered pathways in 1,25(OH)_2_D_3_-treated prostate cancer cells. Those pathways were then combined and merged into one larger network to study the interplay and connections between the pathways. To include more of the differentially expressed genes that are not present in any of the pathways, the network was extended with protein–protein and transcription factor–target interactions to include the first neighbours of the gene products in the pathways. To explore the extended network in more detail, relevant up- and/or down-regulated network modules were identified to highlight the active parts in the network. As a last step, the network was extended with microRNA–target interactions from validated and prediction databases. In this step, the microRNA expression data can be included and combined with the mRNA data and subnetworks of differentially expressed microRNAs and their neighbours can be studied in detail.

### Transcriptomics and microRNA data sets

Statistical analysis performed in Wang’s study revealed that 833 genes were differentially expressed in LNCaP cells treated with 1,25(OH)_2_D_3_. Many of these genes are known to be vitamin D-responsive genes (Krishnan et al. 2004; Peehl et al. 2004; Wang et al. 2005). A complete list of the differentially expressed genes is available in the additional file six of Wang et al. (2011). Four hundred and twenty genes were found to be up-regulated and 413 genes down-regulated in the 1,25(OH)_2_D_3_-treated cells.

Additionally to the transcriptomics data, Wang et al. (2011) examined the effect of 1,25(OH)_2_D_3_ on microRNA expression. They identified nine significantly up-regulated microRNAs in 1,25(OH)_2_D_3_-treated cells with a fold change greater than two. No down-regulated microRNAs were found.

### Pathway analysis

Pathway analysis in PathVisio using the WikiPathways human collection of curated pathways revealed 15 altered pathways in the 1,25(OH)_2_D_3_-treated prostate cancer cells (see Table 1). The results confirm the conclusions of the study by Wang that 1,25(OH)_2_D_3_ affects cell cycle activity. Additionally, the related DNA replication and damage response pathways are changed. Interestingly, there are seven cancer-related pathways that are significantly altered in 1,25(OH)_2_D_3_-treated cancer cells: RB in cancer, gastric cancer networks 1 and 2, integrated pancreatic cancer pathway, integrated breast cancer pathway, integrated cancer pathway and signalling pathways in glioblastoma. Out of 833 differentially expressed genes, the significantly altered pathways contain 73 regulated genes, 14 up-regulated and 59 down-regulated.

**Table 1:** Ranked biological pathways based on *Z* score

Pathway	Positive	Measured	*Z* score	*p* value	Cancer/general
Retinoblastoma (RB) in cancer	35	87	12.63	0.001	Cancer
DNA replication	22	42	11.91	0.001	General
Cell cycle	34	100	11.04	0.001	General
Gastric cancer network 1	15	26	10.44	0.001	Cancer
Histone modifications	25	64	10.44	0.001	General
G1-to-S cell cycle control	23	67	9.12	0.001	General
DNA damage response	15	64	5.4	0.001	General
Gastric cancer network 2	9	30	5.13	0.001	Cancer
ATM signalling pathway	10	38	4.87	0.001	General
Fluoropyrimidine activity	8	32	4.16	0.001	General
Integrated pancreatic cancer pathway	27	195	4.08	0.001	Cancer
Integrated cancer pathway	8	35	3.85	0.005	Cancer
Integrated breast cancer pathway	21	157	3.41	0.002	Cancer
Arylhydrocarbon receptor (AhR) signalling pathway	5	27	2.47	0.015	General
Signalling pathways in glioblastoma	10	82	2.02	0.048	Cancer

Pathway statistics identified 15 significantly altered pathways in 1,25(OH)_2_D_3_-treated prostate cancer cells (*Z* score >1.96, *p* value <0.05 and minimum number of positive genes is five). Besides eight more general cell cycle-related pathways, pathway analysis also revealed seven cancer-specific pathways that are changed. Measured is number of gene products in the pathway that are measured in the data set, and positive is the number of differentially expressed genes in the pathway

The pathway diagrams of all significantly altered pathways are available in Supplementary Material 2.

### Network of interconnected pathways

**Fig. 2:**
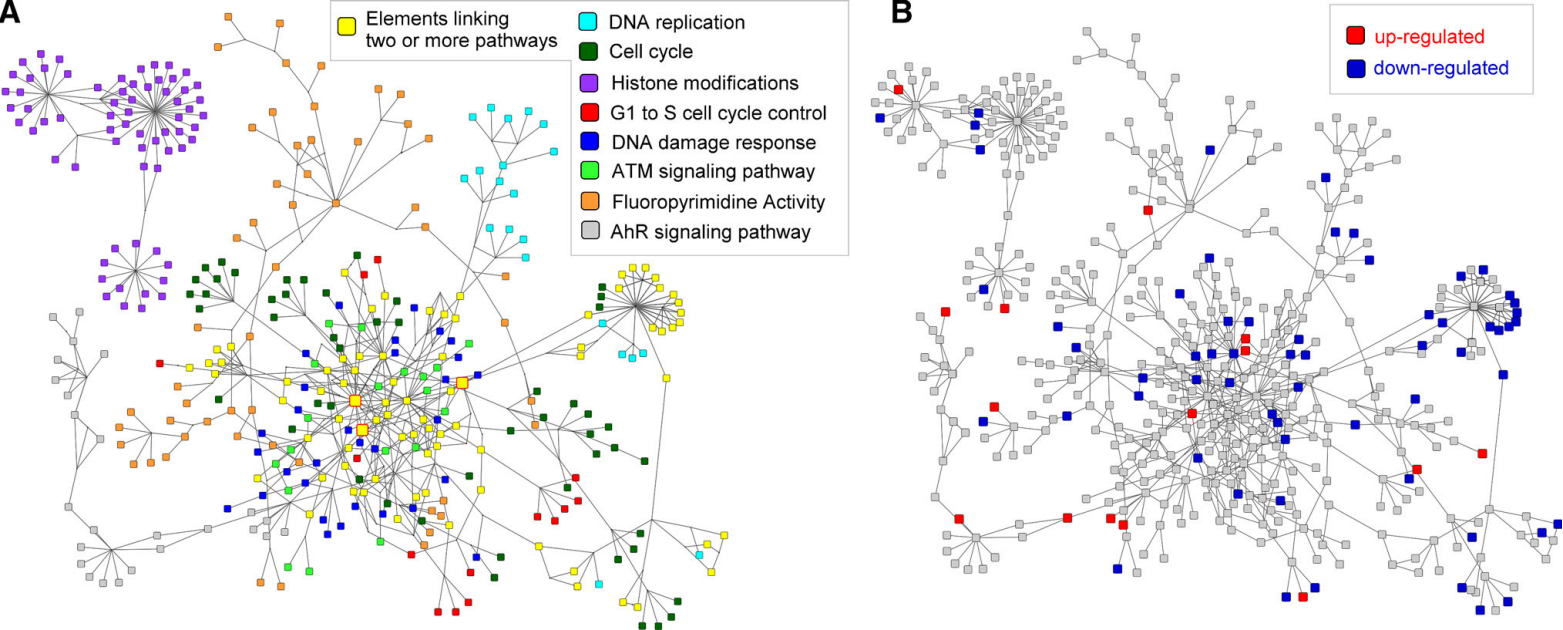
Network of interconnected more general pathways. Pathway analysis revealed eight significantly altered more general pathways in 1,25(OH)_2_D_3_-treated cancer cells. The pathways were merged into one network using the WikiPathways and BridgeDb apps for Cytoscape. **a** The fill colours of the nodes in the network indicate their affiliation with one of the more general pathways. *Yellow* nodes in the network highlight pathway elements linking two or more pathways to each other. **b** Fourteen genes in the network are up-regulated (*red*) and 59 genes are down-regulated (*blue*) in 1,25(OH)_2_D_3_-treated cancer cells

The 15 altered pathways were grouped in a group of explicitly cancer-related pathways and a group of more general (mostly cell cycle related) pathways (see Table 1). The eight more general pathways were merged into one large network containing 503 nodes and 743 edges (see Fig. 2a). The nodes consist of 319 gene products, 28 metabolites and 15 nodes linking to other pathways. The remaining 141 nodes are used to represent groups and complex interactions.

The network of interconnected more general pathways contains 81 nodes that are present in more than one pathway and are therefore linking the different pathways to each other. *TP53*, *CDKN1A* and *CDK2*, three genes known to play important roles in the cell cycle process, are linking five out of eight pathways and therefore are very central nodes in the network. Seven out of eight pathways are connected in one large network. Five of the eight pathways are tightly linked through many shared pathway elements, DNA replication, cell cycle, G1-to-S cell cycle control, DNA damage response and ATM signalling pathways. The AhR signalling pathway connects through four gene products to four of the other pathways, *JUN*, *BAX*, *CDKN1A* and *CDKN1B*. The fluoropyrimidine activity pathway is linked by two nodes to four other pathways, the *TP53* gene product and the apoptosis pathway node. The histone modifications pathway is not connected to any of the other pathways.

The network of interconnected more general pathways contains 73 differentially expressed genes (see Fig. 2b). Fourteen genes in the network are up-regulated in 1,25(OH)_2_D_3_-treated cells. Only one of the up-regulated genes is present in more than one pathway, *ABL1* a gene involved in cell differentiation, cell division, cell adhesion and stress response. Twenty-eight out of 59 down-regulated genes are linking two or more pathways. *CDK1* and *CCNB1* are both significantly down-regulated and present in four out of eight pathways. Both are key players in cell cycle regulation.

Additionally, the seven cancer-related pathways were merged into a network; see Supplementary Material 3. This network contains 540 nodes and 951 edges. Out of 440 gene products, 18 are up-regulated and 66 are down-regulated indicating a down-regulation of cancer pathways in 1,25(OH)_2_D_3_-treated cancer cells. In the following steps, we will only use the network of interconnected more general pathways.

### Network extension

Although 73 differentially expressed genes are present in the network of interconnected more general pathways, many of the differentially expressed genes in the data set are missing. Therefore, the network was extended with known first neighbours of the genes in the pathways to evaluate whether that increases the coverage of the differentially expressed genes found.

First, the network was extended with known protein–protein interactions from the STRING database, and we found 443 additional significantly changed genes that are first neighbours of one of the genes in the pathways. In a next step, we added transcription factor–target interactions from ENCODE and found 67 new differentially expressed genes that are directly linked to one of the genes in the pathways.

The vitamin D-extended network consists of 1013 nodes and 9200 edges. Five hundred and three nodes were from the selected more general pathways (73 differentially expressed), 443 nodes were added as first neighbours from STRING and 67 nodes were extracted from the ENCODE transcription factor-target network. So in total, the network now contains 70 % of the differentially expressed genes (583 out of 833, 345 down-regulated and 238 up-regulated genes); see Supplementary Material 4. The first neighbours also connect the histone modification pathway to the other pathways in the network.

### Active network modules

The vitamin D-extended network comprises all genes in the selected pathways and a large part of the differentially expressed genes. Using the jActiveModules app in Cytoscape, connected small subnetworks (modules) in the vitamin D-extended network were identified in which gene expression was regulated by 1,25(OH)_2_D_3_ treatment. The analysis revealed ten different active modules, the highest scoring module (score = 18.9) contained 193 down-regulated genes. Forty-one nodes in the module were present in the significantly altered pathways.

**Fig. 3:**
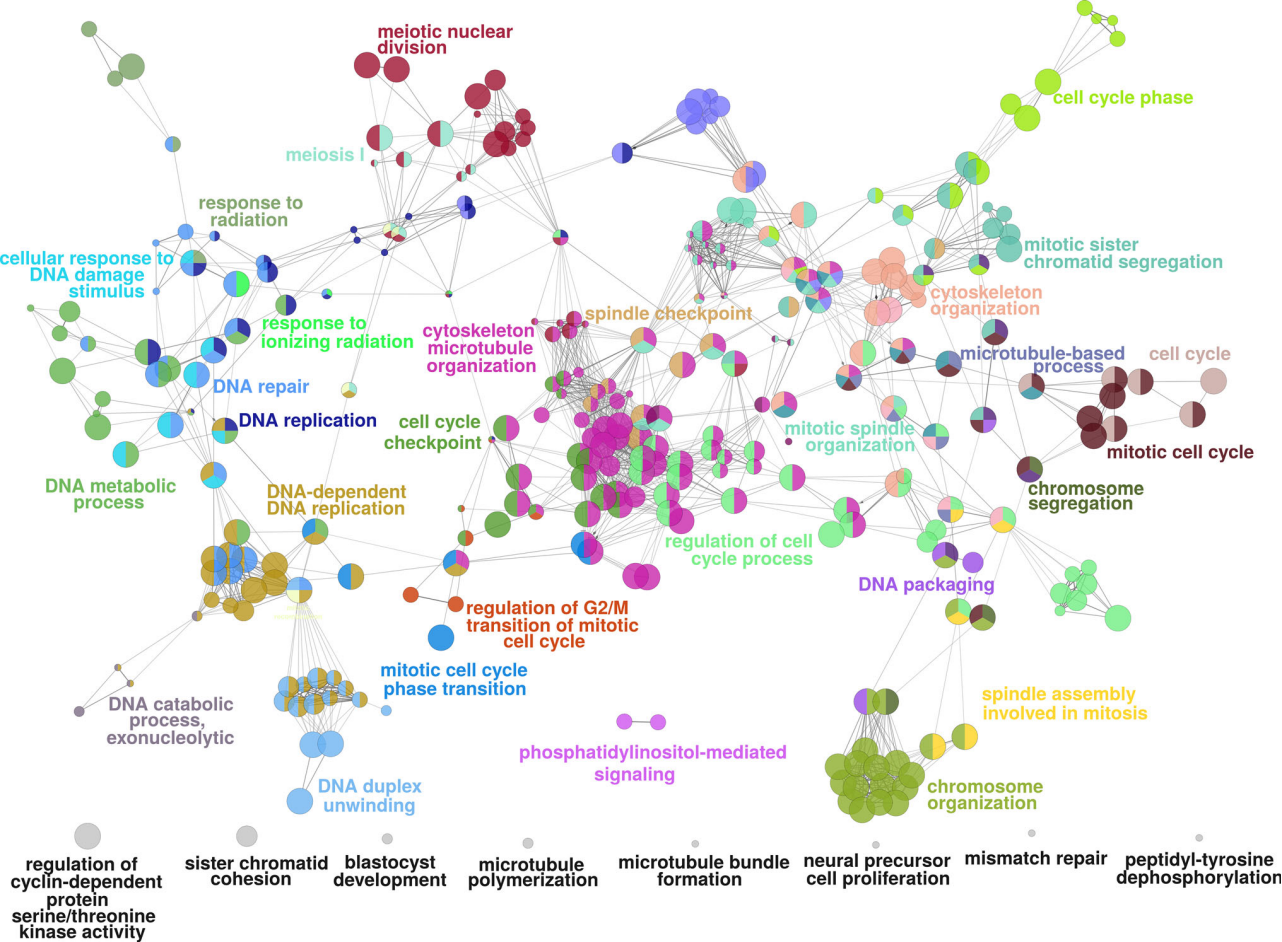
ClueGO network for highest scoring active module. The highest scoring active module in the network with 193 down-regulated genes was identified using the jActiveModules app. Then, the ClueGO app was used to find relevant GO processes, and a network of connected GO terms was created. Each node represents a GO biological process, and the colours represent the GO group. In total, 34 GO groups and 8 GO processes not assigned to a group (shown in *grey*) are present in the network. Per group one representing GO biological process is named in the figure

We then used the Cytoscape app ClueGO to find biological processes in which the genes in the module are involved to identify important functions of the active subnetwork. ClueGO created a network of interconnected GO biological processes based on the similarity of their associated genes (see Fig. 3). Supplementary Material 5 shows all the relevant GO processes and their associated genes in the active module. The network of GO processes contains biological processes involved in DNA processing, cell cycle activity, organelle organization and phosphorylation. This is in accordance with the results of the pathway analysis, which already pointed strongly towards cell cycle-related processes.

### Vitamin D-microRNA network

**Fig. 4:**
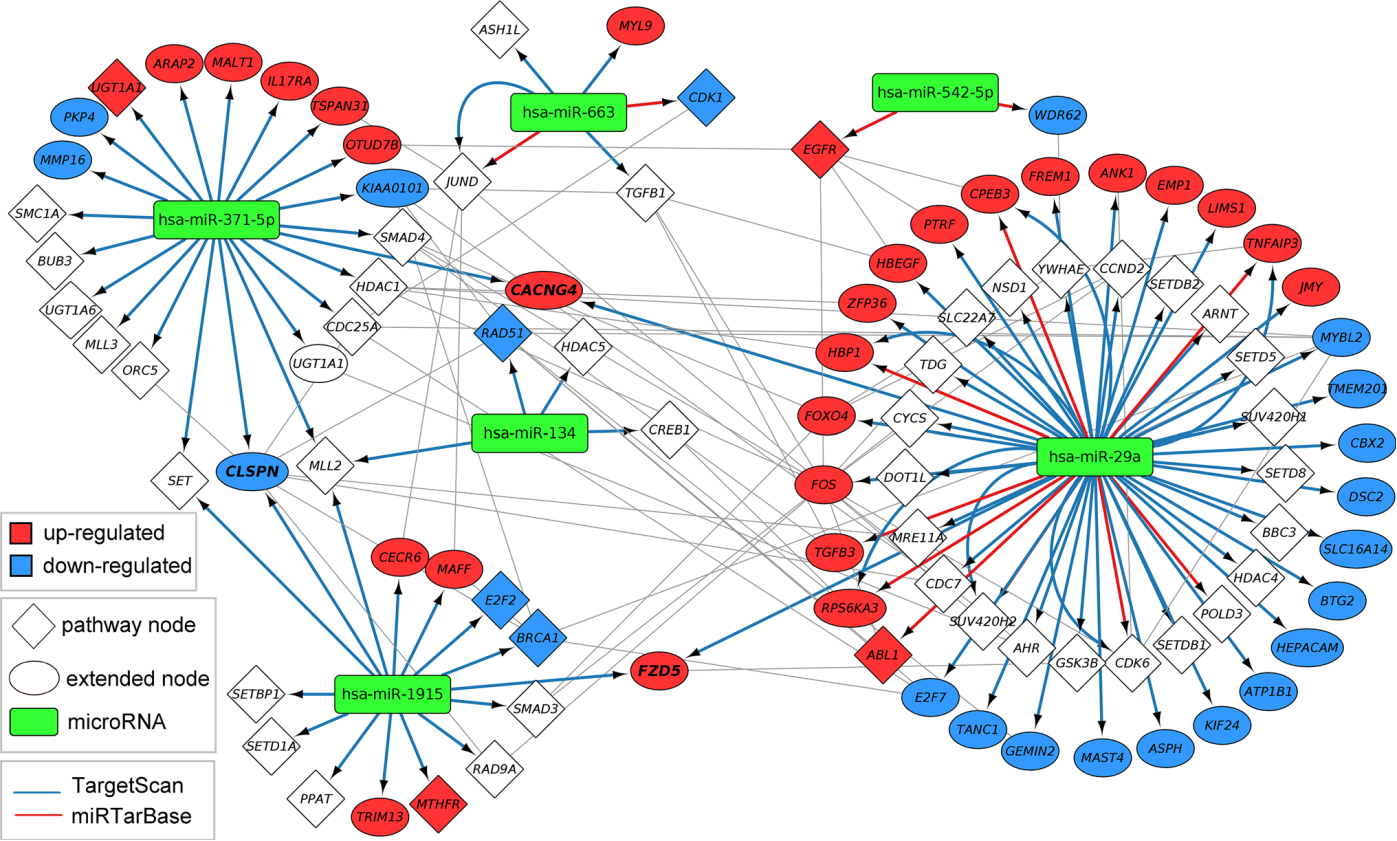
Vitamin D-microRNA network. MicroRNA–target interactions from TargetScan and miRTarBase were added to the vitamin D-extended network. Six out of nine differentially expressed microRNAs in 1,25(OH)_2_D_3_-treated cancer cells were present in the vitamin D-microRNA network. Those six up-regulated microRNAs are highlighted as *green rounded rectangles*. Together they target 96 gene products present in pathways (*diamonds*) and added with protein–protein and transcription factor–gene interactions (*ellipses*). Up-regulated genes are coloured in *red*, and down-regulated genes are coloured in *blue*. The edge colour indicates the source, either TargetScan (*blue*) or miRTarBase (*red*). Seven target interactions are present in both databases

Using the CyTargetLinker app in Cytoscape, a regulatory layer of microRNAs was added to the network. One thousand four hundred and thirty-nine microRNA nodes and 25,886 microRNA–target interactions were found when combining the information from the prediction database TargetScan (version 6.2—23,091 interaction) and the validated database miRTarBase (version 4.5—2795 interactions). Six of the nine differentially expressed microRNAs in the data set were found in this new vitamin D-microRNA network, hsa-miR-29a, hsa-miR-371-5p, hsa-miR-1915, hsa-miR-663, hsa-miR-134 and hsa-miR-542-5p. All six are up-regulated in 1,25(OH)_2_D_3_-treated cancer cells. Consequently, we selected the six up-regulated microRNAs in the network and created a subnetwork with all their targets in the network (see Fig. 4). All microRNAs target genes in the pathways (diamonds) and extended first neighbours (ellipses).

Fifty out of 96 targets are present in the altered pathways (shown as diamonds in Fig. 4), but only seven pathway genes are differentially expressed. Five out of those seven are gene products that link two or more pathways to each other. The six up-regulated microRNAs target genes in all eight more general pathways.

Except for hsa-miR-134, all other microRNAs target up- and down-regulated genes. Interactions for hsa-miR-371-5p, hsa-miR-1915 and hsa-miR-134 are only present in the prediction database TargetScan. The microRNA hsa-miR-542-5p has only interactions from the validated database miRTarBase. The microRNAs hsa-miR-29a and hsa-miR-663 have interactions from TargetScan and miRTarBase; seven of them are even present in both databases, like hsa-miR-663 → JUND or hsa-miR-29a → CPEB3.

### Highlighting biological results

The presented integrative network-based analysis provides a useful framework to study the effects of 1,25(OH)_2_D_3_ on a process level as well as to define new hypotheses about the mechanisms of 1,25(OH)_2_D_3_ in prostate cancer. In this section, we would like to highlight several biological results to showcase the relevance of the approach described.

#### G1-to-S phase transition

The pathway analysis revealed that the G1-to-S cell cycle pathway was among the altered pathways after 1,25(OH)_2_D_3_ treatment in LNCaP cells. This pathway had a *Z* score of 9.12 (*p* value = 0.001) and contained 23 differentially expressed genes, 21 down-regulated genes and 2 up-regulated genes. The pathway diagram visualizing the differentially expressed genes is present in the Supplementary Material 2. Yang and Burnstein (2003) showed that 1,25(OH)_2_D_3_ could block the transition of the LNCaP cells from the G1-to-S phase. They showed that after 1,25(OH)_2_D_3_ treatment the p27 protein levels increased and that the nuclear localization of Cdk2 decreased, resulting in a reduced Cdk2 activity. The gene expression in the present study confirms the inhibition of the G1-to-S transition. Among the down-regulated genes in our pathway is cyclin *CCNE2*. *CCNE2* forms a complex with and regulates Cdk2. The decreased expression of *CCNE2* after 1,25(OH)_2_D_3_ treatment might lead to a reduced Cdk2 activity, and in this way, it might prevent G1-to-S transition.

#### Cancer-related pathways

Pathway databases like WikiPathways also store disease-specific pathways. In our analysis, the pathway statistics result in PathVisio also showed several cancer-related pathways, which clearly indicates that the 1,25(OH)_2_D_3_ treatment affects cancer-related processes. The pathway diagrams of the integrated cancer and *RB* in cancer pathways in the Supplementary Material 2 clearly show that several cancer-related processes are down-regulated in 1,25(OH)_2_D_3_-treated prostate cancer cells. This confirms several earlier studies and suggests that prostate cancer patients may benefit from vitamin D treatment (Hatse et al. 2012; Shui and Giovannucci 2014).

#### Cytoskeleton organization

The GO analysis in the highest regulated module from the vitamin D-extended network showed that the differentially expressed genes in the module are present in cell cycle-related GO processes. Interestingly, several processes are associated with the cytoskeleton organization. In our pathway analysis, the processes related to cytoskeleton organization are not significantly changed by 1,25(OH)_2_D_3_. However, Max et al. (2014) recently studied gene expression in rat muscle of newborns after maternal treatment with vitamin D. They found that cell cycle and cytoskeletal processes are altered after such a treatment. These processes are similar to what we found in prostate cancer cells using GO analysis. Moreover, our approach demonstrates that extending the network of interconnected pathways with differentially expressed genes that are not in the altered pathways can identify processes that are not significant in pathway analysis.

#### VDR target analysis

Transcriptional regulation is a central process in humans, and VDR is a member of the nuclear receptor family of transcription factors. VDR is activated by 1,25(OH)_2_D_3_ and can form a heterodimer with RXR to regulate transcription of many different target genes. In an extensive literature search, we identified 178 genes that are known to be regulated by VDR.

In 1,25(OH)_2_D_3_-treated prostate cancer cells, we found 21 VDR target genes that are differentially up-regulated and two that are differentially down-regulated; see Supplementary Material 1. *CYP24A1* is the most up-regulated gene in the experiment (FC = 56), and this mitochondrial protein is responsible for the degradation of 1,25(OH)_2_D_3_. An increase in expression after 1,25(OH)_2_D_3_ treatment is therefore expected.

##### VDR targets in the network of interconnected pathways

In the network of interconnected general pathways that has been built from the significantly altered pathways, ten of the gene products are known VDR targets. Most of the targets are present in the G1-to-S cell cycle control pathway. No targets have been found in two out of eight pathways: histone modifications and fluoropyrimidine activity. Only two of the targets are up-regulated (*CDKN2B and IGFBP1*), and two are down-regulated (*CDKN2C* and *CDKN2D*). Two out of three genes that connect five different pathways are known targets of VDR (*CDKN1A* and *CDK2*), clearly showing that they play an important role in response to 1,25(OH)_2_D_3_ treatment.

There are only three VDR targets in the network of interconnected cancer-related pathways, *MYC*, *CDKN2B* and *CDKN2C.* Interestingly, all three genes are differentially expressed in 1,25(OH)_2_D_3_-treated prostate cancer cells. *CDKN2B* is up-regulated, and *MYC* and *CDKN2C* are down-regulated.

##### VDR targets in the extended network

After the extension with interactions from STRING and ENCODE, 17 of the differentially expressed VDR targets are present in the network. Six of the differentially expressed VDR targets are not present in the extended network, *CLMN*, *DND1*, *ORM1*, *ORM2*, *SULT1C2* and *STEAP4*. *ORM1* and *ORM2*, for example, encode for the alpha-1-acid glycoprotein, an acute-phase plasma protein that has been reported as a possible prognostic factor of survival in cancer patients (Bruno et al. 2003).

The VDR analysis showed that several known VDR targets are significantly changed in 1,25(OH)_2_D_3_-treated LNCaP cells and that they are mostly up-regulated. However, also the expression of other genes is affected by the 1,25(OH)_2_D_3_ treatment. This could indicate that they are regulated either indirectly or independently of VDR. Including knowledge on VDR targets added an additional regulatory level to our network analysis.

#### MicroRNA targets

In the vitamin D-microRNA network, microRNA–target interactions from TargetScan and miRTarBase were added to the vitamin D-extended network. Five genes are targeted by more than one microRNA, *CLSPN*, *FZD5*, *CACNG4*, *SET* and *MLL2*. *CLSPN* is down-regulated, *FZD5* and *CACNG4* are up-regulated, and *SET* and *MLL2* are not differentially expressed in 1,25(OH)_2_D_3_-treated cancer cells. *CLSPN* is an important checkpoint regulator in the cell cycle and is known to be down-regulated by 1,25(OH)_2_D_3_ treatment (Verlinden et al. 2007). This is also shown in the vitamin D-microRNA network. *FZD5* (Frizzled5), a receptor for Wnt proteins, is known to be affected by 1,25(OH)_2_D_3_ treatment (Doroudi et al. 2014). *CACNG4* is a calcium channel, and even though there is no direct relation to vitamin D known, Wiki-pi, a Web server of annotated human protein–protein interactions to aid the discovery of protein function, shows that the known interaction partners of *CACNG4* are enriched in the GO term “response to vitamin D” (Orii and Ganapathiraju 2012).

### Features of our network-based integrative analysis

In this study, we showed how pathway- and network-based approaches can be used to analyse and integrate different omics measurements. Pathway analysis is a powerful tool to analyse experimental data and puts the data into a biological context. Pathways are not closed systems, but they interact and influence each other. In our analysis, we merged all the significantly altered pathways into one network to be able to get a more complete view of the changes in the system. Additionally, it is possible to see pathway elements that link different pathways to each other.

Biological pathway databases like WikiPathways, Reactome and KEGG are constantly growing, but there is still a lot of information missing. Therefore, it is important to combine pathway knowledge with information about binary interactions like protein–protein or transcription factor–gene interactions. There are many online databases storing such interactions, and by integrating them in the network, more of the changes in the data set can be included in the analysis. Instead of 73 differentially expressed genes in the pathways, we were able to include 583 differentially expressed genes after extending the network with protein–protein interactions from STRING and transcription factor–gene interactions from ENCODE.

There are many different network algorithms that can be used to investigate the vitamin D-extended network. In our analysis, we selected two different approaches to demonstrate the power of network analysis. A commonly used method to find relevant parts in the network is the identification of active modules. Active modules are groups of connected genes that are activated or repressed. Finding active modules is a hard problem and jActiveModules involves random sampling, so the calculation results might slightly differ between two runs. In the vitamin D-extended network, active modules were identified based on the gene expression in 1,25(OH)_2_D_3_-treated prostate cancer cells compared to non-treated cancer cells. The highest scoring module of down-regulated genes seemed to be very robust, with only a few genes added or removed. Functional enrichment of the genes in the selected module in GO biological processes was determined using the ClueGO app. A network of interconnected GO processes showed processes involved in DNA processing, cell cycle activity, organelle organization and phosphorylation. In the network, GO processes are linked and similar or deviating GO processes become apparent.

In a second network analysis step, we included microRNA–target interactions to make it possible to study the microRNA and mRNA expression data together. The microRNA–target interactions were added with the CyTargetLinker app. We selected one validated (miRTarBase) and one prediction (TargetScan) database for this step. Six out of nine differentially expressed microRNAs were found in the network. It is now possible to extract the subnetwork of the six microRNAs and all their targets. This enables the user to study this relevant part of the network in more detail.

Biological pathways consist of different types of gene products and metabolites. The different types of gene product types (DNA, RNA and protein) are often combined in one entity, but the different types can also be mapped to each other. That enables the integration and visualization of omics data sets from different omics technologies (van Iersel et al. 2014). However, biological pathways often do not contain all the different regulatory elements like microRNAs and transcription factors to keep the pathways concise and understandable. By using network analysis, we are now able to combine and integrate the different interaction and regulation levels and analyse and visualize different omics data set, like mRNA and microRNA, together.

All the approaches in this analysis are highly generic and can be used for different data sets in human or other species. The extension systems of software tools like PathVisio and Cytoscape through plugins or apps allow users to apply a variety of different methods within the same framework which facilitates the analysis process and saves a lot of time.

### Challenges and future directions

In the present study, the effect of 1,25(OH)_2_D_3_ in prostate cancer cells was investigated in vitro. Although it shows how gene and microRNA expression in human prostate cancer cells can be influenced by vitamin D, it would be of interest to determine the whole-body effect of vitamin D. Prostate cancer patients could benefit from vitamin D treatment, and therefore, clinical studies investigating the effect of vitamin D supplement on the global gene and microRNA expression level in various tissues could shed a new light on the vitamin D health effect. The approach in the present study can be applied in a similar way to in vivo vitamin D studies.

Finally, in addition to gene and microRNA expression data, other types of data could be included into a network-based analysis. By combining other characteristics like age, gender, genetic variation, vitamin D blood levels before and after supplementation or tumour size, patients could be divided into subgroups which could respond differently to the vitamin D treatment. Knowledge on the biological processes or parts of the processes that are affected in patients could tell whether vitamin D supplementation had the desired effect or not.

## Conclusion

In this analysis, we demonstrated how to integrate mRNA and microRNA expression in 1,25(OH)_2_D_3_-treated prostate cancer cells in a network-based analysis. By combining biological pathways, protein–protein interactions, transcription factor–gene interactions and microRNA regulation data, it is possible to study the effect of 1,25(OH)_2_D_3_ treatment on a more systematic level.

Our approach showed that 1,25(OH)_2_D_3_ in LNCaP cells affects gene expression in cell cycle-related processes including the G1-to-S phase transition. Interestingly, also several cancer-related processes are mostly down-regulated in the 1,25(OH)_2_D_3_-treated cells.

Moreover, the expression of several VDR target genes, which were among the 178 identified VDR target genes, were up-regulated after 1,25(OH)_2_D_3_ treatment, for example *CYP24A1*, the most up-regulated gene in the experiment (FC = 56.22), that is mitochondrial protein responsible for the degradation of 1,25(OH)_2_D_3_.

Finally, the gene targets of the significantly expressed microRNAs after 1,25(OH)_2_D_3_ treatment could be identified by adding microRNA regulation. Six of the nine differentially expressed microRNAs target genes in the extended network, including *CLSPN*, an important checkpoint regulator in the cell cycle that is down-regulated, and *FZD5*, a receptor for Wnt proteins that is up-regulated.

Taken together, this work also shows that the network-based tools, PathVisio and Cytoscape, enable a straightforward, in-depth and biologically meaningful integrative analysis of mRNA and microRNA expression.

## Electronic supplementary material

Supplementary material 1 (PDF 567 kb)

Supplementary material 2 (PDF 2486 kb)

Supplementary material 3 (PDF 672 kb)

Supplementary material 4 (PDF 724 kb)

Supplementary material 5 (PDF 207 kb)
