# Supplementary material for: Integrative network-based analysis of mRNA and microRNA expression in 1,25-dihydroxyvitamin D3-treated cancer cells
Source: Genes Nutr. 2015 Aug 15;10(5):35. doi: 10.1007/s12263-015-0484-0 (PMC4537452; doi:10.1007/s12263-015-0484-0)
Supplement: Supplementary file 1 — Supplementary material 1 (PDF 567 kb) [file 12263_2015_484_MOESM1_ESM.pdf]

## Supplementary Material 1: VDR target gene analysis

**Article Title:** Integrative network-based analysis of mRNA and microRNA expression in 1,25-dihydroxyvitamin D3-treated cancer cells
**Journal name:** Genes & Nutrition
**Author Names:** Martina Kutmon, Susan L Coort, Kim de Nooijer, Claire Lemmens, Chris T Evelo
**Email address:** martina.kutmon@maastrichtuniversity.nl

---

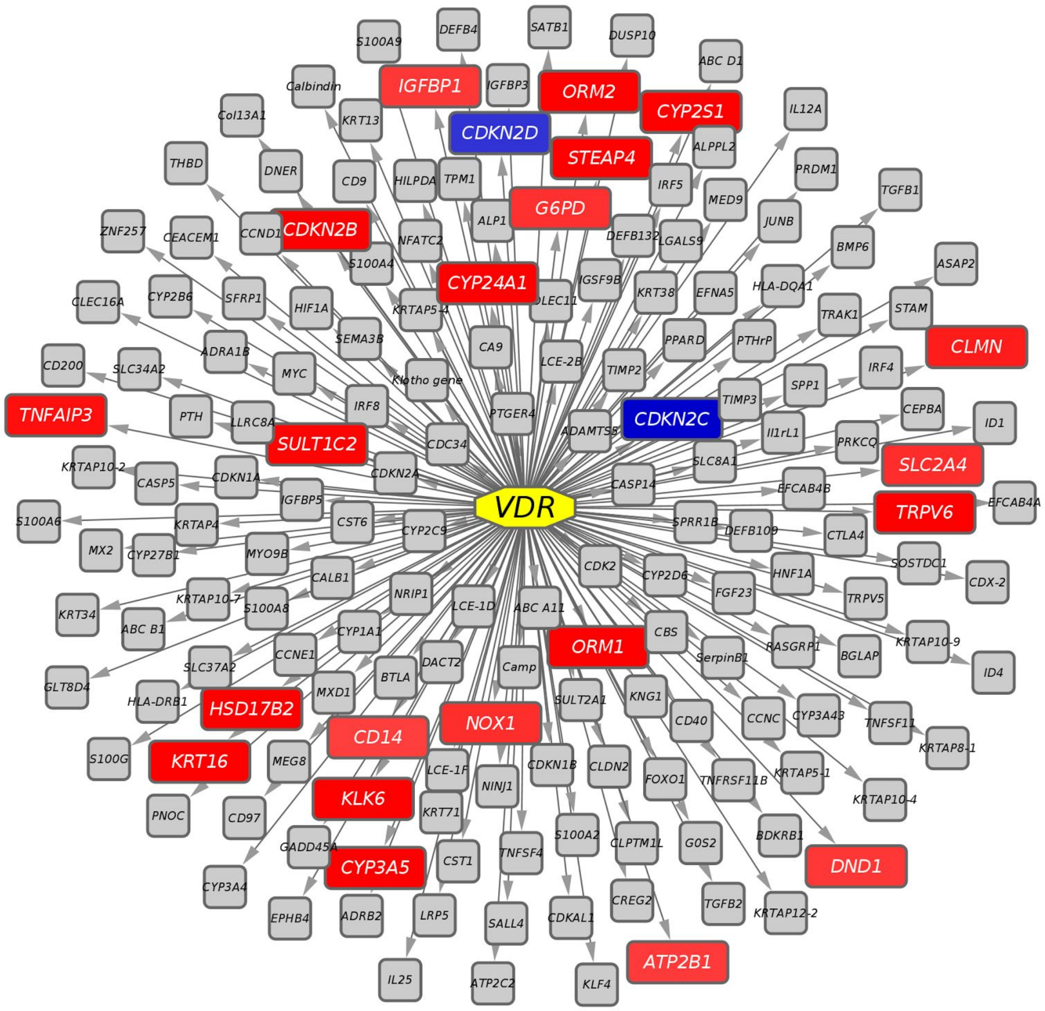


**Figure S1:** 178 VDR targets and their gene expression in 1,25(OH)$_2$D$_3$-treated cancer cells (red indicates up-regulated, blue indicates down-regulation).

The list with VDR target genes was manually compiled from the experimental data presented in the 25 research papers below.

1. Bouillon, Roger, et al. "Vitamin D and energy homeostasis - of mice and men." *Nature Reviews Endocrinology* 10.2 (2014): 79-87.
2. Bunce, Chris M., and Moray J. Campbell, eds. *Nuclear receptors: current concepts and future challenges*. Vol. 8. Springer Science & Business Media, 2010.
3. Calberg, C. "Target genes of vitamin D: spatio-temporal interaction of chromatin, VDR, and response elements." *Vitamin D (third ed.), Elsevier Academic Press, San Diego, CA* (2011): 211-226.

4. Carlberg, Carsten, and Sabine Seuter. "A genomic perspective on vitamin D signaling." *Anticancer research* 29.9 (2009): 3485-3493.
5. Carlberg, Carsten, Sabine Seuter, and Sami Heikkinen. "The first genome-wide view of vitamin D receptor locations and their mechanistic implications."*Anticancer research* 32.1 (2012): 271-282.
6. Carlberg, Carsten, et al. "Primary vitamin D target genes allow a categorization of possible benefits of vitamin D3 supplementation." *PLoS One* 8.7 (2013): e71042.
7. Chatterjee, Bandana, Ibtissam Echchgadda, and Chung Seog Song. "Vitamin D receptor regulation of the steroid/bile acid sulfotransferase SULT2A1." *Methods in enzymology* 400 (2005): 165-191.
8. Chen, Hankui, et al. "Vitamin D directly regulates Mdm2 gene expression in osteoblasts." *Biochemical and biophysical research communications* 430.1 (2013): 370-374.
9. Deeb, Kristin K., Donald L. Trump, and Candace S. Johnson. "Vitamin D signalling pathways in cancer: potential for anticancer therapeutics." *Nature Reviews Cancer* 7.9 (2007): 684-700.
10. Drissi, Hicham, et al. "1, 25-(OH) 2-vitamin D3 suppresses the bone-related Runx2/Cbfa1 gene promoter." *Experimental cell research* 274.2 (2002): 323-333.
11. Drocourt, Lionel, et al. "Expression of CYP3A4, CYP2B6, andCYP2C9 Is Regulated by the Vitamin D Receptor Pathway in Primary Human Hepatocytes."*Journal of Biological Chemistry* 277.28 (2002): 25125-25132.
12. Echchgadda, Ibtissam, et al. "Dehydroepiandrosterone sulfotransferase is a target for transcriptional induction by the vitamin D receptor." *Molecular pharmacology* 65.3 (2004): 720-729.
13. Feldman, D., J. W. Pike, and J. S. Adams. "Vitamin D. Vol 2." (2011).
14. Goeman, Frauke, et al. "VDR primary targets by genome-wide transcriptional profiling." *The Journal of steroid biochemistry and molecular biology* 143 (2014): 348-356.
15. Haussler, Mark R., et al. "Vitamin D receptor (VDR)-mediated actions of 1α, 25 (OH) 2 vitamin D 3: genomic and non-genomic mechanisms." *Best Practice & Research Clinical Endocrinology & Metabolism* 25.4 (2011): 543-559.
16. Haussler, Mark R., et al. "Molecular mechanisms of vitamin D action." *Calcified tissue international* 92.2 (2013): 77-98.
17. Matilainen, Merja, et al. "Regulation of multiple insulin-like growth factor binding protein genes by 1α, 25-dihydroxyvitamin D3." *Nucleic acids research* 33.17 (2005): 5521-5532.
18. Pike, J. Wesley, Mark B. Meyer, and Kathleen A. Bishop. "Regulation of target gene expression by the vitamin D receptor-an update on mechanisms."*Reviews in Endocrine and Metabolic Disorders* 13.1 (2012): 45-55.
19. Ramagopalan, Sreeram V., et al. "A ChIP-seq defined genome-wide map of vitamin D receptor binding: associations with disease and evolution." *Genome research* 20.10 (2010): 1352-1360.
20. Ryynänen, Jussi, et al. "Changes in vitamin D target gene expression in adipose tissue monitor the vitamin D response of human individuals." *Molecular nutrition & food research* 58.10 (2014): 2036-2045.
21. Ryynänen, Jussi, et al. "Gene regulatory scenarios of primary 1, 25-dihydroxyvitamin D3 target genes in a human myeloid leukemia cell line."*Cancers* 5.4 (2013): 1221-1241.
22. Seuter, Sabine, Jussi Ryynänen, and Carsten Carlberg. "The ASAP2 gene is a primary target of 1, 25-dihydroxyvitamin D 3 in human monocytes and macrophages." *The Journal of steroid biochemistry and molecular biology* 144 (2014): 12-18.
23. Wang, Tian-Tian, et al. "Large-scale in silico and microarray-based identification of direct 1, 25-dihydroxyvitamin D3 target genes." *Molecular endocrinology*19.11 (2005): 2685-2695.
24. Wilfinger, Julia, et al. "Primary vitamin D receptor target genes as biomarkers for the vitamin D 3 status in the hematopoietic system." *The Journal of nutritional biochemistry* 25.8 (2014): 875-884.
25. Yasunami, Yoko, et al. "C-jun N-terminal kinase modulates 1, 25-dihydroxyvitamin D3-induced cytochrome P450 3A4 gene expression." *Drug metabolism and disposition* 32.7 (2004): 685-688.

**Table S1:** 178 VDR target genes

| Gene name | NCBI Gene ID | References (Pubmed ID) | DE |
|---|---|---|---|
| *ABCA11* | 79963 | 22782502, 21872797 | |
| *ABCB1* | 5243 | 22782502, 21872797 | |
| *ABCD1* | 215 | 22782502, 21872797 | |
| *ADAMTS5* | 11096 | 24247221 | |
| *ADRA1B* | 147 | 22782502, 21872797 | |
| *ADRB2* | 154 | 22782502, 21872797 | |
| *ALOX5* | 240 | Feldman (2011), 19667142, 22213316, 20736230 | |
| *ALP1* | 248 | 22782502, 21872797 | |
| *ALPPL2* | 251 | 22782502, 21872797 | |
| *ASAP2* | 8853 | 23999061 | |
| *ATP2B1* | 490 | 21870057 | UP |
| *ATP2C2* | 9914 | 22782502, 21872797 | |
| *BDKRB1* | 623 | 24726990 | |
| *BGLAP* | 632 | 17721433, 22782502, 21872797 | |
| *BMP6* | 654 | 22782502, 21872797 | |
| *BTLA* | 151888 | 22213316 | |
| *CA9* | 768 | 22782502, 21872797 | |
| *CALB1* | 12307 | 21870057 | |
| *CAMP* | 820 | 16002434, 21872797, 22782502 | |
| *CASP14* | 23581 | 22782502, 16002434, 21872797 | |
| *CASP5* | 838 | 16002434 | |
| *CBS* | 875 | 21872797, 22782502 | |
| *CCNC* | 892 | Feldman (2011), 19667142, 22213316, 17721433, 20736230 | |
| *CCND1* | 595 | Feldman (2011) | |
| *CCNE1* | 898 | Feldman (2011) | |
| *CD14* | 929 | 24854954, 23923049 | UP |
| *CD200* | 4345 | 22213316 | |
| *CD40* | 958 | 20736230 | |
| *CD9* | 928 | 24247221 | |
| *CD97* | 976 | 24854954 | |
| *CDC34* | 997 | 22782502, 21872797 | |
| *CDK2* | 1017 | Feldman (2011) | |
| *CDKAL1* | 54901 | 20736230 | |
| *CDKN1A* | 1026 | Feldman (2011), 19667142, 22213316, 17721433, 21872797, 24202443, 22782502 | |
| *CDKN1B* | 1027 | Feldman (2011), 19667142 | |
| *CDKN2A* | 1029 | Feldman (2011) | |
| *CDKN2B* | 1030 | Feldman (2011) | UP |
| *CDKN2C* | 1031 | Feldman (2011) | DOWN |
| *CDKN2D* | 1032 | Feldman (2011) | DOWN |
| *CDX-2* | 1045 | 22782502, 21872797 | |
| *CEACAM1* | 634 | 21872797 | |
| *CEBPA* | 1050 | 22782502, 21872797 | |
| *CLDN2* | 9075 | 22782502, 21872797 | |
| *CLEC16A* | 23274 | 20736230 | |
| *CLMN* | 79789 | 16002434 | UP |
| *CLPTM1L* | 81037 | 20736230 | |
| *COL13A1* | 1305 | 16002434 | |
| *COLEC11* | 78989 | 22782502, 21872797 | |

| | | | |
|---|---|---|---|
| *CREG2* | 200407 | 22782502, 21872797 | |
| *CST1* | 1469 | 22782502, 21872797 | |
| *CST6* | 1474 | 16002434 | |
| *CTLA4* | 1493 | 20736230 | |
| *CYP1A1* | 1543 | 22782502, 21872797 | |
| *CYP24A1* | 1591 | Feldman (2011), 19667142, 22213316, 17721433, 24726990, 21872797, 22782502 | UP |
| *CYP27B1* | 1594 | Feldman (2011), 19667142, 22213316, 23923049, 17721433, 24726990, 22782502 | |
| *CYP2B6* | 1555 | 11991950 | |
| *CYP2C9* | 1559 | 11991950 | |
| *CYP2D6* | 1565 | 22782502, 21872797 | |
| *CYP2S1* | 29785 | 22782502, 21872797 | UP |
| *CYP3A4* | 1576 | 15205382, 11991950, 21872797, 22782502, 16002434 | |
| *CYP3A43* | 64816 | 22782502, 21872797 | |
| *CYP3A5* | 1577 | 22782502, 21872797 | UP |
| *DACT2* | 168002 | 22782502, 21872797 | |
| *DEFB109* | 100286963 | 22782502, 21872797 | |
| *DEFB132* | 400830 | 22782502, 21872797 | |
| *DEFB4* | 1673 | 16002434 | |
| *DND1* | 373863 | 22782502, 21872797 | UP |
| *DNER* | 92737 | 22782502, 21872797 | |
| *DUSP10* | 11221 | 24975273, 22782502, 21872797 | |
| *EFCAB4A* | 283229 | 22782502, 21872797 | |
| *EFCAB4B* | 84766 | 22782502, 21872797 | |
| *EFNA5* | 1946 | 22782502, 21872797 | |
| *EPHB4* | 2050 | 22782502, 21872797 | |
| *FGF23* | 8074 | Feldman (2011), 21872797, 22782502, 21870057 | |
| *FOXO1* | 2308 | 16002434, 21872797, 22782502 | |
| *G0S2* | 50486 | 24202443 | |
| *G6PD* | 2539 | 22782502, 21872797 | UP |
| *GADD45A* | 1647 | Feldman (2011), 17721433, 16002434 | |
| *GLT8D4* | 727936 | 22782502, 21872797 | |
| *HIF1A* | 3091 | 16002434 | |
| *HILPDA* | 29923 | 24247221 | |
| *HLA-DQA1* | 3117 | 20736230 | |
| *HLA-DRB1* | 3123 | 20736230 | |
| *HNF1A* | 6927 | 22782502, 21872797 | |
| *HSD17B2* | 3294 | 16002434 | UP |
| *ID1* | 3397 | 22782502, 21872797 | |
| *ID4* | 3400 | 24726990 | |
| *IGFBP1* | 3484 | 16186133, Feldman (2011), 19667142, 22213316 | UP |
| *IGFBP3* | 3486 | 16186133, Feldman (2011), 19667142, 22213316, 24726990 | |
| *IGFBP5* | 3488 | 16186133, Feldman (2011), 19667142, 22213316 | |
| *IGSF9B* | 22997 | 22782502, 21872797 | |
| *IL12A* | 3592 | 24726990 | |
| *IL1RL1* | 9173 | 16002434, 24726990 | |
| *IL25* | 64806 | 22782502, 21872797 | |
| *IRF4* | 3662 | 20736230 | |
| *IRF5* | 3663 | 20736230 | |
| *IRF8* | 3394 | 22782502, 21872797 | |
| *JUNB* | 3726 | 22782502, 21872797 | |
| *KL* | 9365 | 21872797, 22782502 | |

| | | | |
|---|---|---|---|
| *KLF4* | 9314 | 24726990 | |
| *KLK6* | 5653 | 16002434 | UP |
| *KNG1* | 3827 | 22782502, 21872797 | |
| *KRT13* | 3860 | 22782502, 21872797 | |
| *KRT16* | 3868 | 22782502, 21872797 | UP |
| *KRT34* | 3885 | 22782502, 21872797 | |
| *KRT38* | 8687 | 22782502, 21872797 | |
| *KRT71* | 112802 | 22782502, 21872797 | |
| *KRTAP10-2* | 386679 | 22782502, 21872797 | |
| *KRTAP10-4* | 386672 | 22782502, 21872797 | |
| *KRTAP10-7* | 386675 | 22782502, 21872797 | |
| *KRTAP10-9* | 386676 | 22782502, 21872797 | |
| *KRTAP12-2* | 353323 | 22782502, 21872797 | |
| *KRTAP4* | 85285 | 22782502, 21872797 | |
| *KRTAP5-1* | 387264 | 22782502, 21872797 | |
| *KRTAP5-4* | 387267 | 22782502, 21872797 | |
| *KRTAP8-1* | 337879 | 22782502, 21872797 | |
| *LCE-1D* | 353134 | 22782502, 21872797 | |
| *LCE-1F* | 353137 | 22782502, 21872797 | |
| *LCE-2B* | 26239 | 22782502, 21872797 | |
| *LGALS9* | 3965 | 22782502, 21872797 | |
| *LRP5* | 4041 | 21872797, Feldman (2011), 19667142, 22213316, 22782502 | |
| *LRRC25* | 126364 | 22213316 | |
| *MED9* | 55090 | 22782502, 21872797 | |
| *MEG8* | 79104 | 22782502, 21872797 | |
| *MX2* | 4600 | 22782502, 21872797 | |
| *MXD1* | 4084 | 16002434 | |
| *MYC* | 4609 | 24202443, Feldman (2011), 16002434 | |
| *MYO9B* | 4650 | 22213316 | |
| *NFATC2* | 4773 | 22782502, 21872797 | |
| *NINJ1* | 4814 | 22213316 | |
| *NOX1* | 27035 | 22782502, 21872797 | UP |
| *NRIP1* | 8204 | 24975273, 24854954 | |
| *ORM1* | 5004 | 22782502, 21872797 | UP |
| *ORM2* | 5005 | 22782502, 21872797 | UP |
| *PNOC* | 5368 | 22782502, 21872797 | |
| *PPARD* | 5467 | Feldman (2011), 19667142 | |
| *PRDM1* | 639 | 20736230 | |
| *PRKCQ* | 5588 | 20736230 | |
| *PTGER4* | 5734 | 20736230 | |
| *PTH* | 5741 | Feldman (2011), 23923049, 17721433, 24726990, 21872797, 22782502 | |
| *PTHLH* | 5744 | 21872797, 22782502 | |
| *RASGRP1* | 10125 | 20736230 | |
| *S100A2* | 6273 | 16002434, 22782502 | |
| *S100A4* | 6275 | 22782502, 21872797 | |
| *S100A6* | 6277 | 22782502, 21872797 | |
| *S100A8* | 6279 | 22782502, 16002434, 21872797 | |
| *S100A9* | 6280 | 22782502, 21872797 | |
| *S100G* | 795 | 21870057 | |
| *SALL4* | 57167 | 22782502, 21872797 | |
| *SATB1* | 6304 | 24726990 | |

| | | | |
|---|---|---|---|
| *SEMA3B* | 7869 | 16002434 | |
| *SERPINB1* | 1992 | 16002434 | |
| *SFRP1* | 6422 | 22782502, 21872797 | |
| *SLC2A4* | 6517 | 24247221 | UP |
| *SLC34A2* | 10568 | Feldman (2011), 21870057 | |
| *SLC37A2* | 219855 | 24854954, 22213316 | |
| *SLC8A1* | 6546 | 21870057 | |
| *SOSTDC1* | 25928 | 21872797, 22782502 | |
| *SPP1* | 6696 | 21872797, 22782502 | |
| *SPRR1B* | 6699 | 22782502, 21872797 | |
| *STAM* | 8027 | 20736230 | |
| *STEAP4* | 79689 | 24247221 | UP |
| *SULT1C2* | 6819 | 22782502, 21872797 | UP |
| *SULT2A1* | 6822 | 14978251, 16399349, 22782502 | |
| *TGFB1* | 7040 | 24247221 | |
| *TGFB2* | 7042 | 22782502, 21872797 | |
| *THBD* | 7056 | 24975273, 24247221, 22213316, 23923049 | |
| *TIMP2* | 7077 | 22782502, 21872797 | |
| *TIMP3* | 7078 | 22782502, 21872797 | |
| *TNFAIP3* | 7128 | 20736230 | UP |
| *TNFRSF11B* | 4982 | 21870057 | |
| *TNFSF11* | 8600 | Feldman (2011), 19667142, 22213316, 21872797, 21870057, 22782502 | |
| *TNFSF4* | 7292 | 20736230 | |
| *TPM1* | 7168 | 24247221 | |
| *TRAK1* | 22906 | 24975273 | |
| *TRPV5* | 56302 | 16002434, 21870057 | |
| *TRPV6* | 55503 | Feldman (2011), 24726990, 16002434, 22782502, 21872797 | UP |
| *ZNF257* | 113835 | 22782502, 21872797 | |
