# Supplementary material for: Integrative network-based analysis of mRNA and microRNA expression in 1,25-dihydroxyvitamin D3-treated cancer cells
Source: Genes Nutr. 2015 Aug 15;10(5):35. doi: 10.1007/s12263-015-0484-0 (PMC4537452; doi:10.1007/s12263-015-0484-0)
Supplement: Supplementary file 2 — Supplementary material 2 (PDF 2486 kb) [file 12263_2015_484_MOESM2_ESM.pdf]

Supplementary Material 2: Pathway diagrams

Article Title: Integrative network-based analysis of mRNA and microRNA expression in 1,25-dihydroxyvitamin D3-treated cancer cells

Journal name: Genes & Nutrition

Author Names: Martina Kutmon, Susan L Coort, Kim de Nooijer, Claire Lemmens, Chris T Evelo

Email address: martina.kutmon@maastrichtuniversity.nl

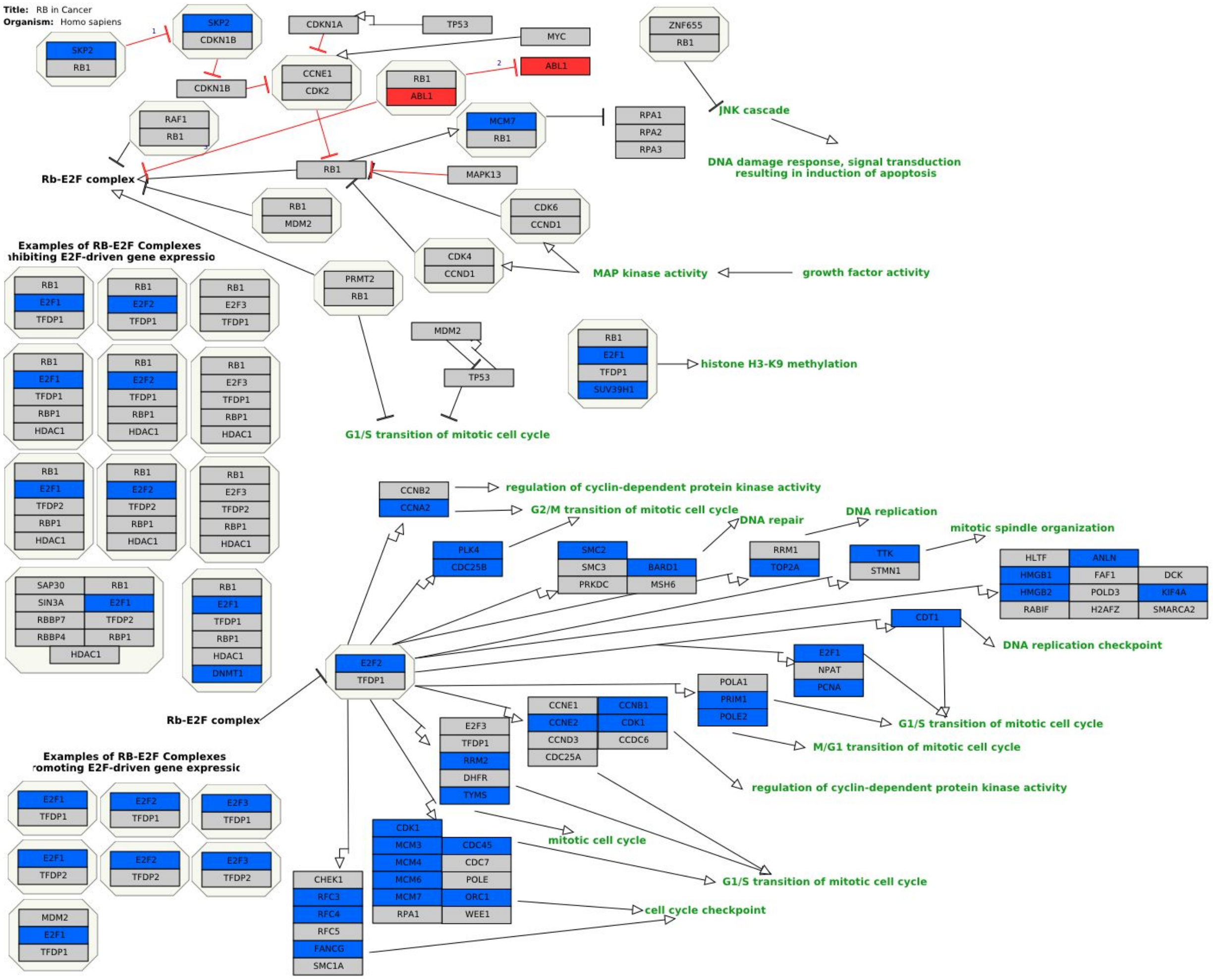

Figure S2A: RB in cancer (http://wikipathways.org/index.php/Pathway:WP2446, revision 78573)
red = significantly up-regulated, blue = significantly down-regulated, z-score = 12.63

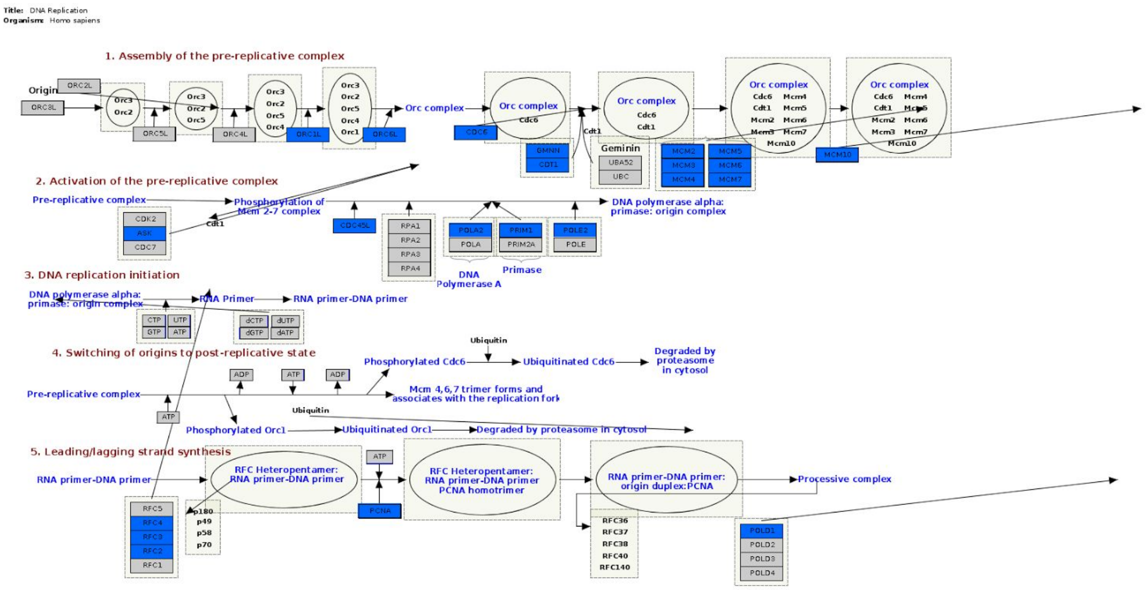

Figure S2B: DNA replication (http://wikipathways.org/index.php/Pathway:WP466, revision 76196)
red = significantly up-regulated, blue = significantly down-regulated, z-score = 11.91

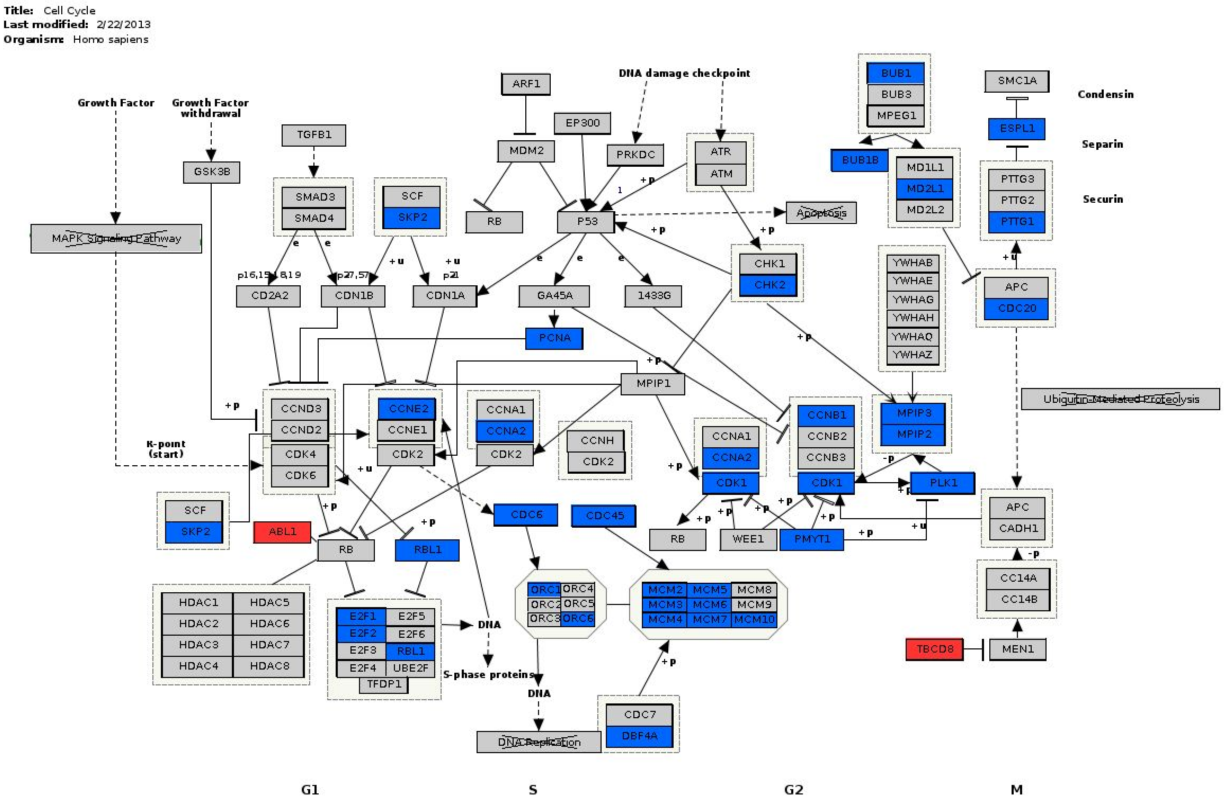

Figure S2C: Cell cycle (http://wikipathways.org/index.php/Pathway:WP179, revision 70629)
red = significantly up-regulated, blue = significantly down-regulated, z-score = 11.04

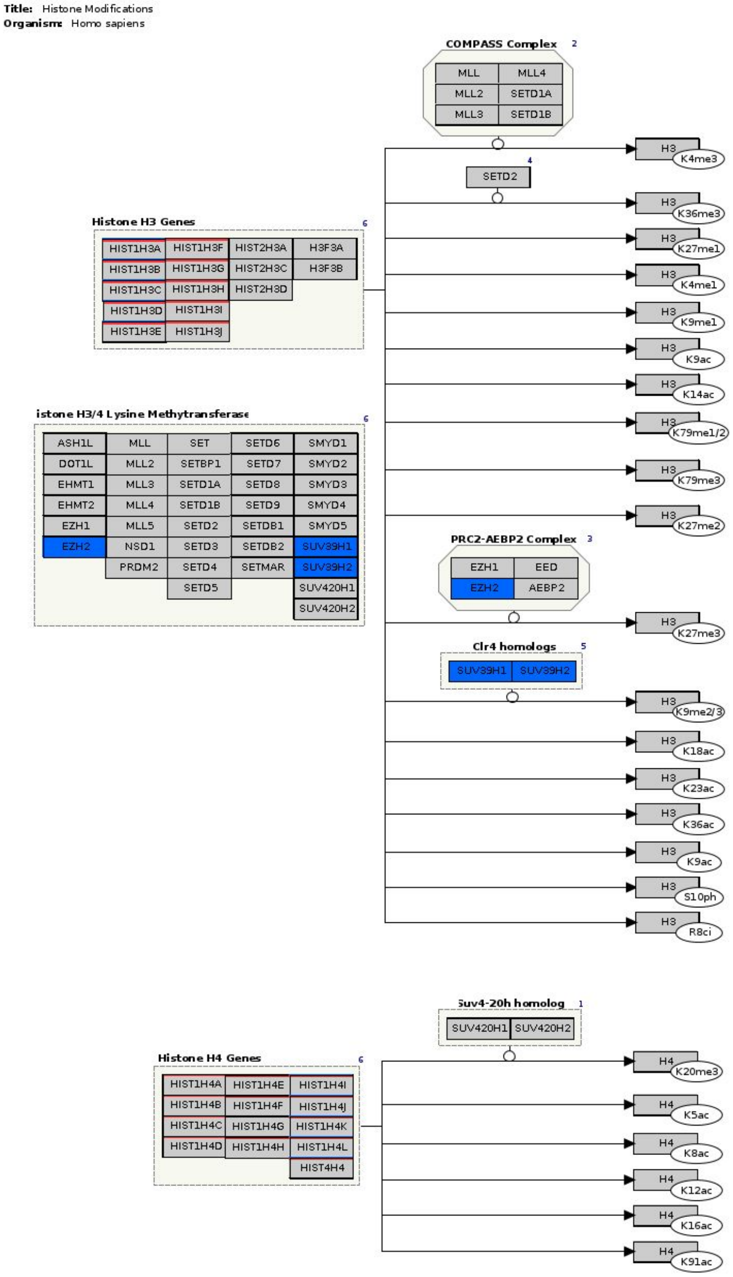

Figure S2D: Histone modifications (http://wikipathways.org/index.php/Pathway:WP2369, revision 69927) red = significantly up-regulated, blue = significantly down-regulated, z-score = 10.44

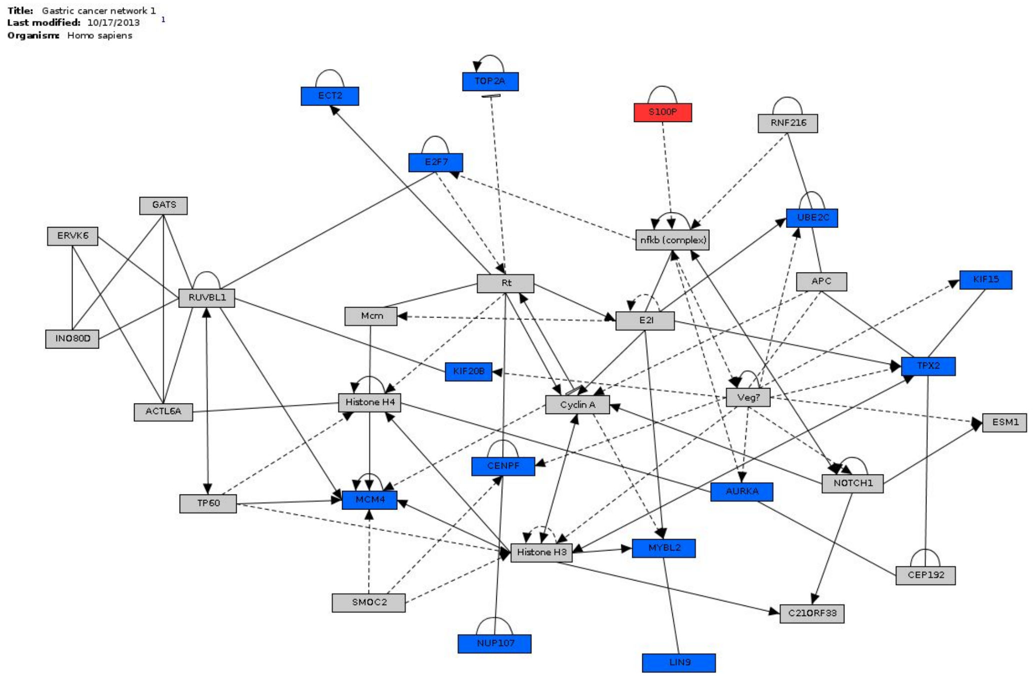

Figure S2E: Gastric cancer network 1 (http://wikipathways.org/index.php/Pathway:WP2361, revision 77608) red = significantly up-regulated, blue = significantly down-regulated, z-score = 10.44

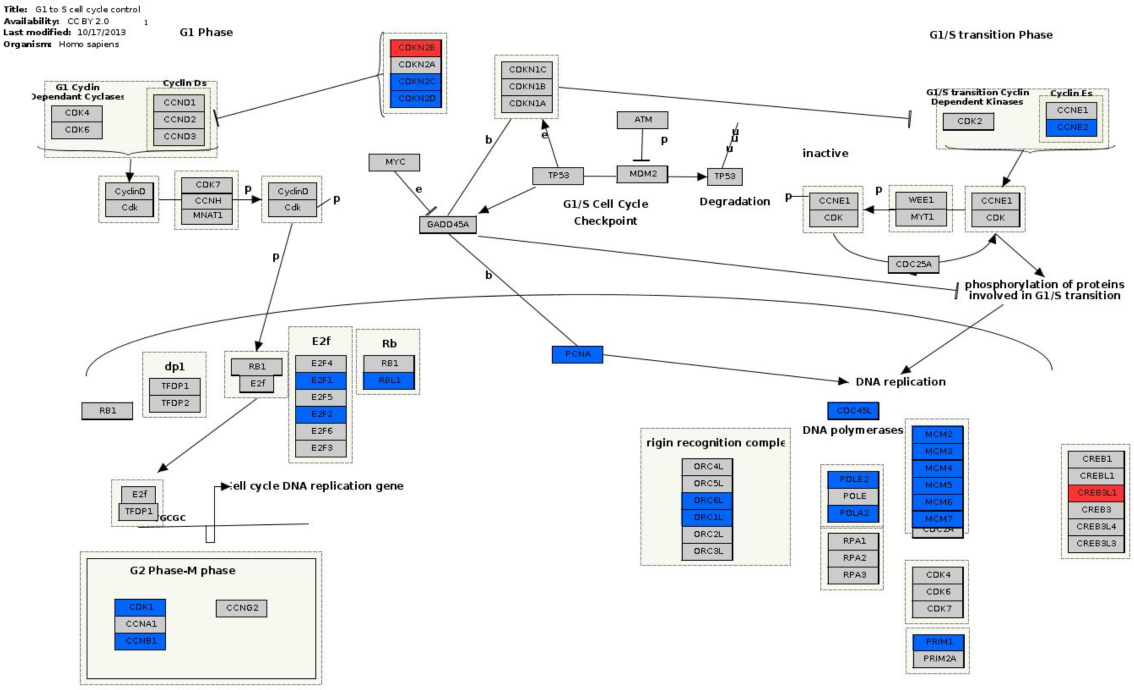

Figure S2F: G1 to S cell cycle control (http://wikipathways.org/index.php/Pathway:WP45, revision 71377) red = significantly up-regulated, blue = significantly down-regulated, z-score = 9.12

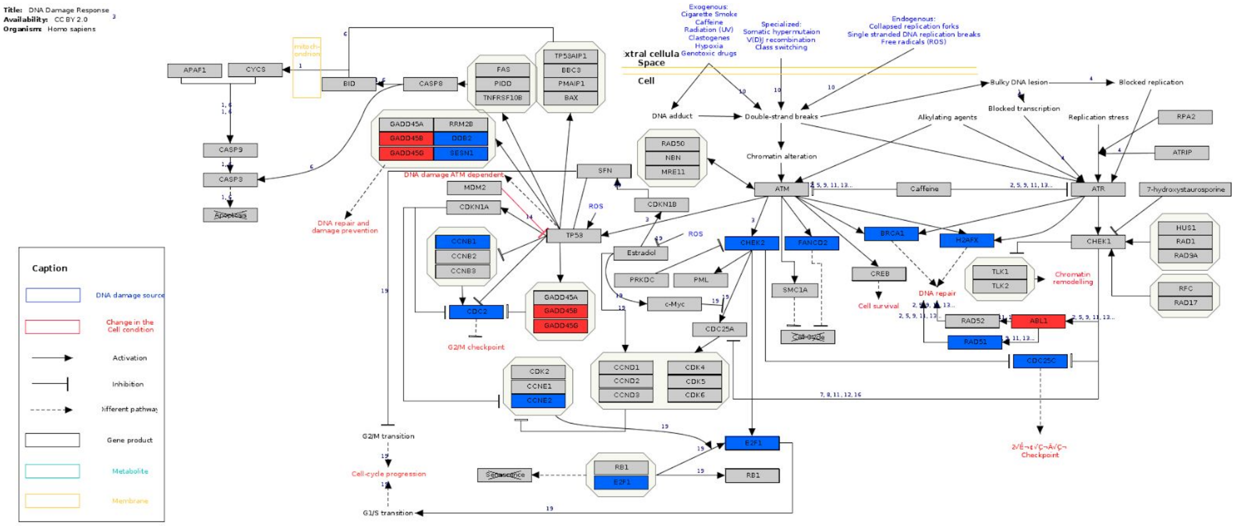

Figure S2G: DNA damage response (http://wikipathways.org/index.php/Pathway:WP707, revision 78527)
red = significantly up-regulated, blue = significantly down-regulated, z-score = 5.40

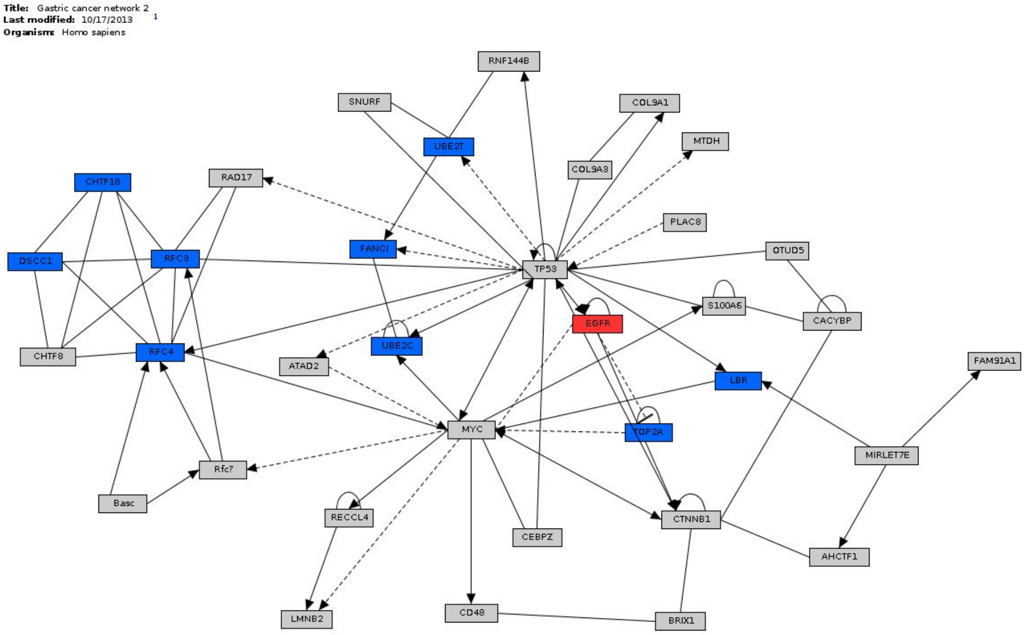

Figure S2H: Gastric cancer network 2 (http://wikipathways.org/index.php/Pathway:WP2363, revision 76329)
red = significantly up-regulated, blue = significantly down-regulated, z-score = 5.13

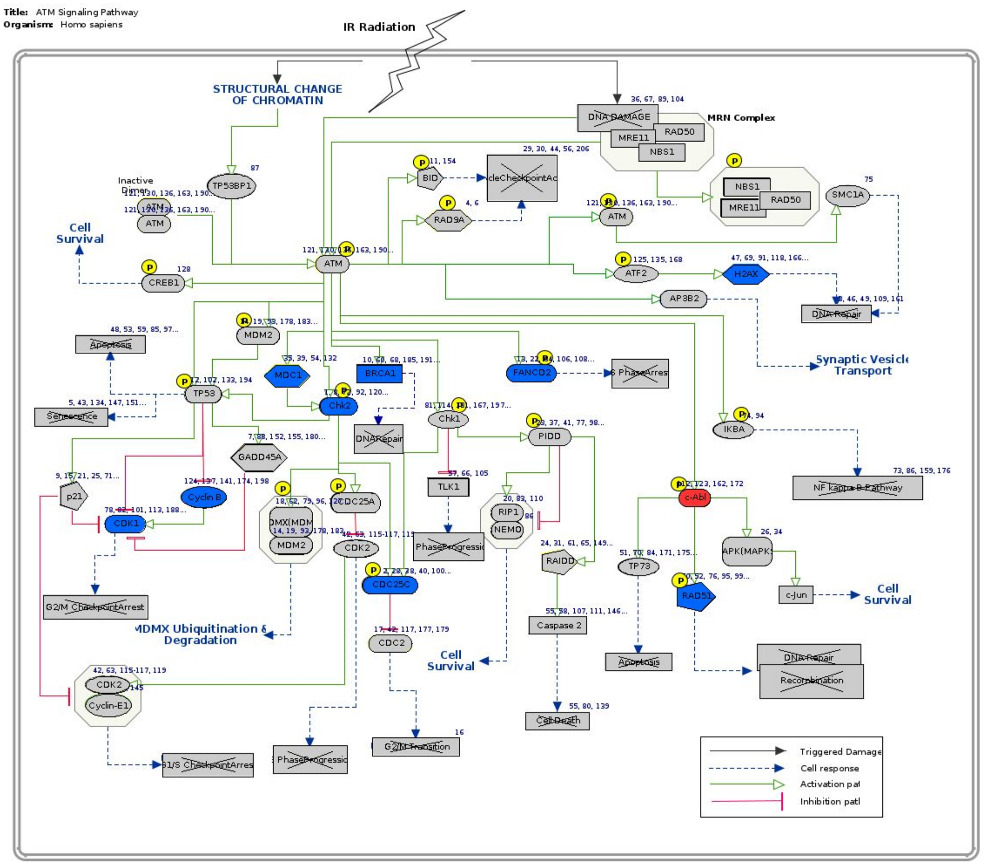

Figure S2I: ATM signaling pathway (http://wikipathways.org/index.php/Pathway:WP2516, revision 78531) red = significantly up-regulated, blue = significantly down-regulated, z-score = 4.87

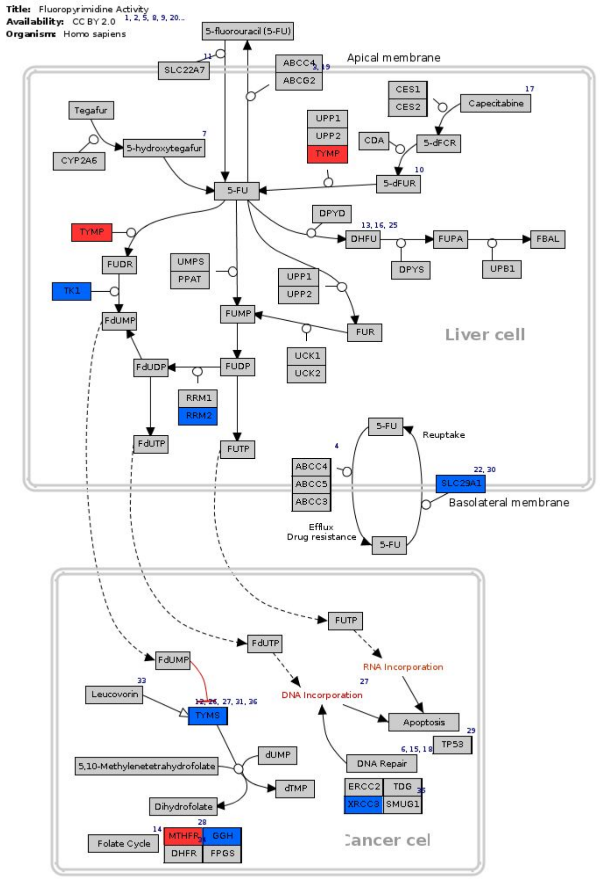

Figure S2J: Fluoropyrimidine Activity (http://wikipathways.org/index.php/Pathway:WP1601, revision 78516) red = significantly up-regulated, blue = significantly down-regulated, z-score = 4.16

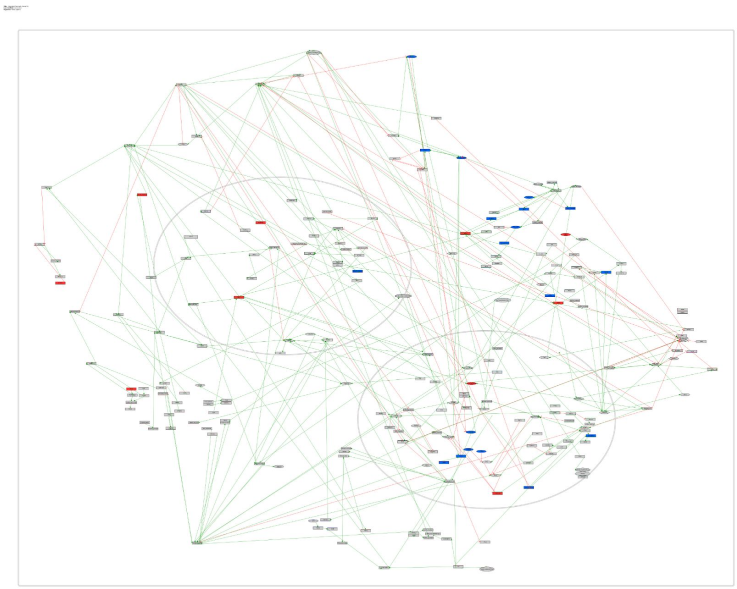

Figure S2K: Integrated Pancreatic Cancer Pathway (http://wikipathways.org/index.php/Pathway:WP2377, revision 71228), red = significantly up-regulated, blue = significantly down-regulated, z-score = 4.08

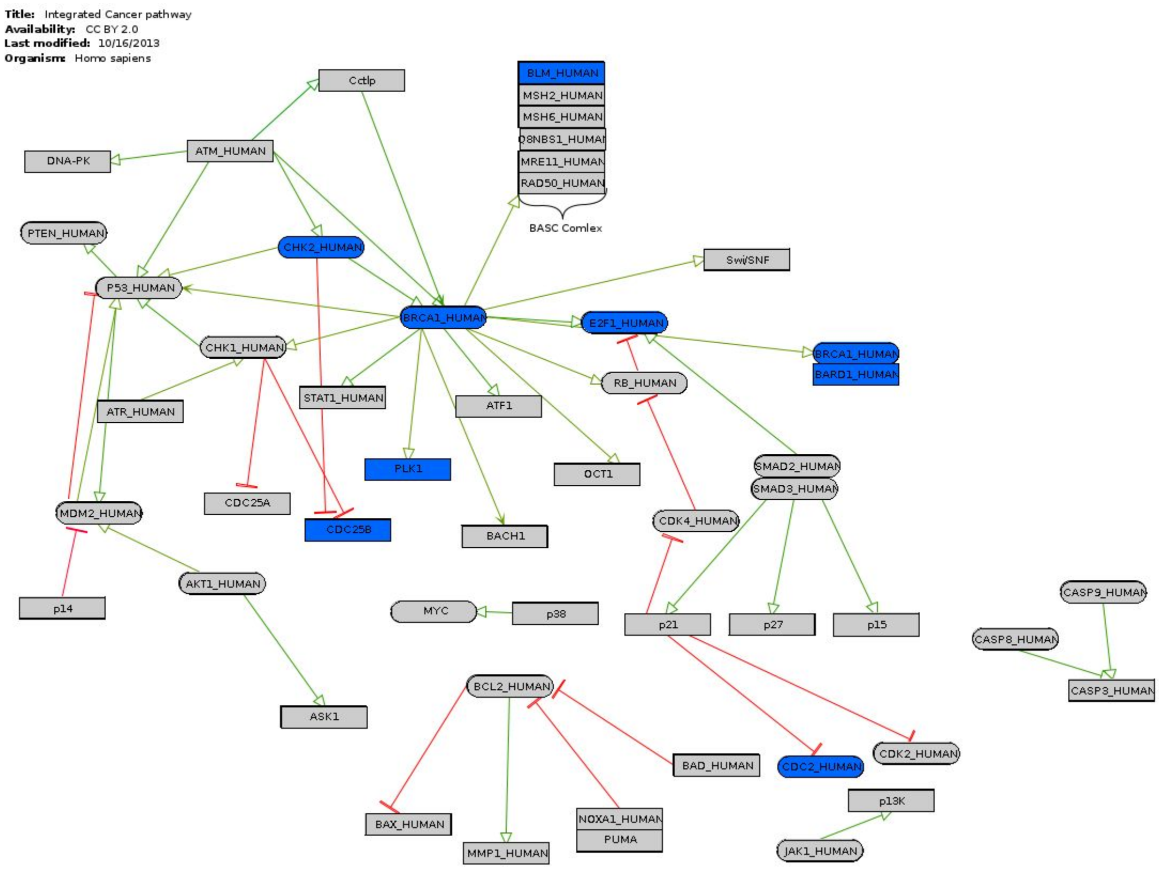

Figure S2L: Integrated Cancer pathway (http://wikipathways.org/index.php/Pathway:WP1971, revision 71249) red = significantly up-regulated, blue = significantly down-regulated, z-score = 3.85

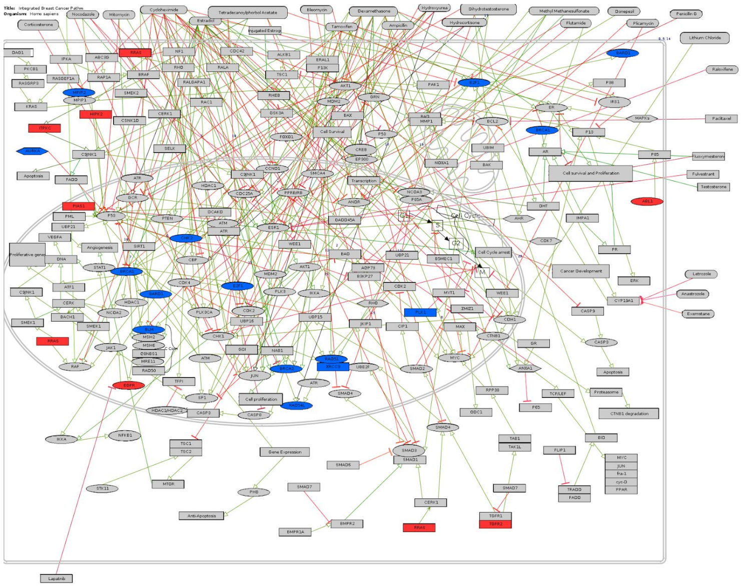

Figure S2M: Integrated Breast Cancer Pathway (http://wikipathways.org/index.php/Pathway:WP1984, revision 72732), red = significantly up-regulated, blue = significantly down-regulated, z-score = 3.41

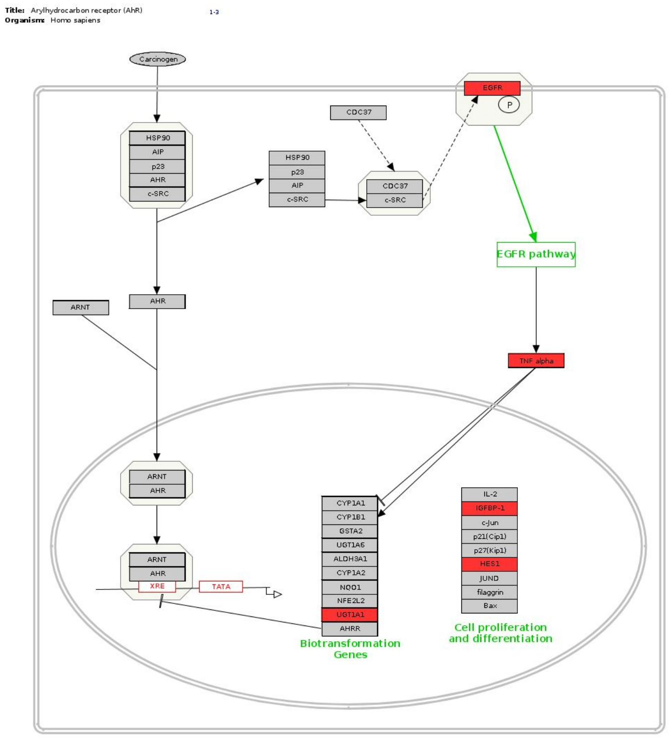

Figure S2N: Arylhydrocarbon receptor (AhR) signaling pathway (http://wikipathways.org/index.php/Pathway:WP2100, revision 74081)
red = significantly up-regulated, blue = significantly down-regulated, z-score = 2.47

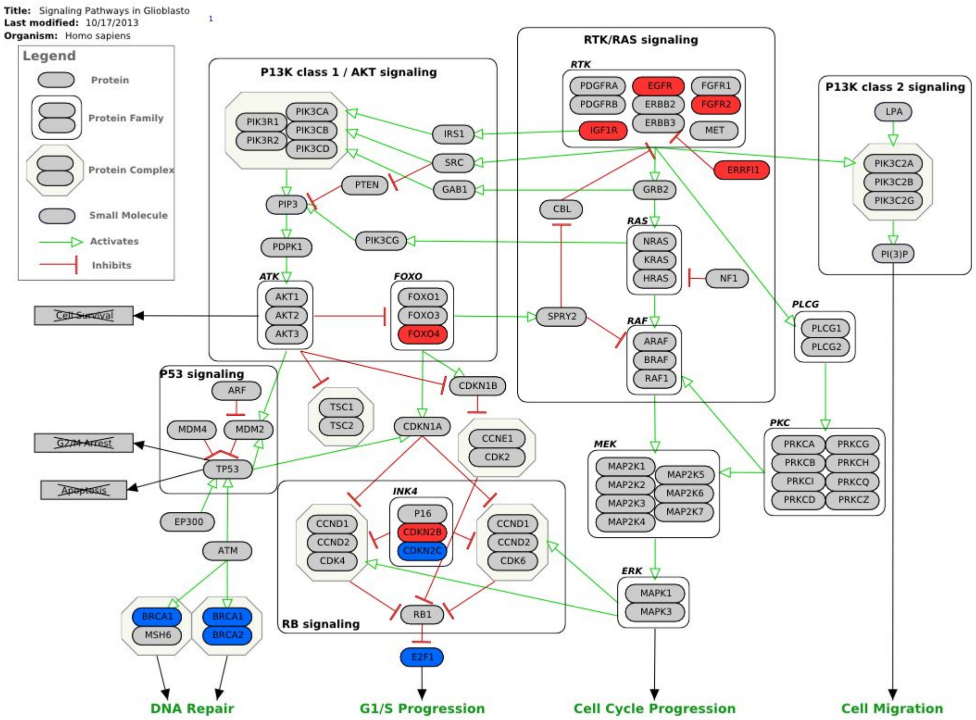

Figure S2O: Signaling Pathways in Glioblastoma (http://wikipathways.org/index.php/Pathway:WP2261, revision 78522), red = significantly up-regulated, blue = significantly down-regulated, z-score = 2.02
