# Supplementary material for: Integrative network-based analysis of mRNA and microRNA expression in 1,25-dihydroxyvitamin D3-treated cancer cells
Source: Genes Nutr. 2015 Aug 15;10(5):35. doi: 10.1007/s12263-015-0484-0 (PMC4537452; doi:10.1007/s12263-015-0484-0)
Supplement: Supplementary file 3 — Supplementary material 3 (PDF 672 kb) [file 12263_2015_484_MOESM3_ESM.pdf]

Supplementary Material 3: Network of interconnected cancer related pathways

Article Title: Integrative network-based analysis of mRNA and microRNA expression in 1,25-dihydroxyvitamin D3-treated cancer cells
Journal name: Genes & Nutrition
Author Names: Martina Kutmon, Susan L Coort, Kim de Nooijer, Claire Lemmens, Chris T Evelo
Email address: martina.kutmon@maastrichtuniversity.nl

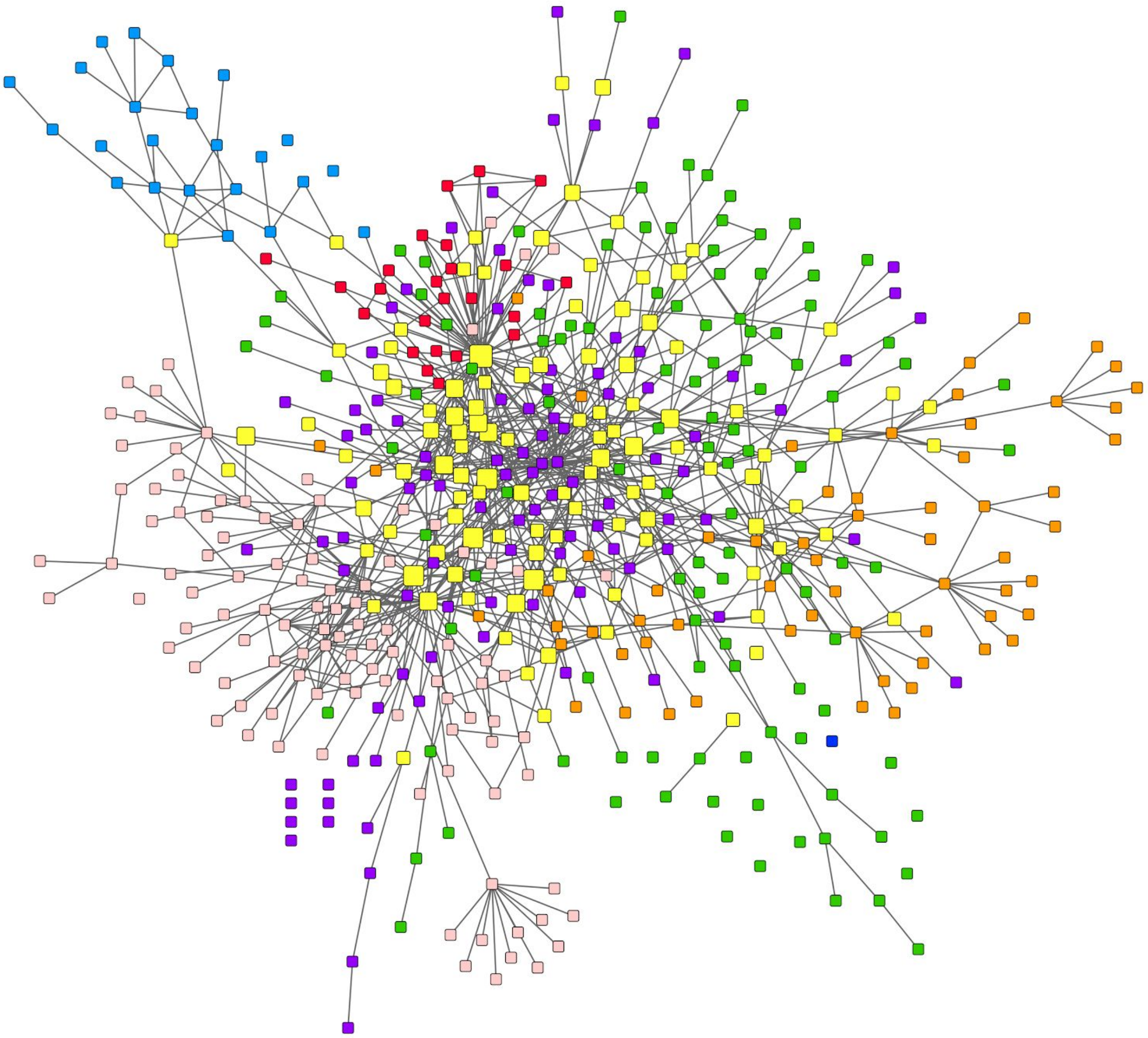

Figure S3A: Pathways highlighted in cancer-related network

Pathway analysis revealed seven significantly altered cancer-related pathways in 1,25(OH)$_2$D$_3$-treated cancer cells (RB in Cancer, Gastric cancer network 1, Gastric cancer network 2, Integrated Pancreatic Cancer Pathway, Integrated Cancer Pathway, Integrated Breast Cancer Pathway, Signaling Pathways in Glioblastoma). The pathways were merged into one network using the WikiPathways and BridgeDb apps for Cytoscape. The fill color of the nodes in the network indicate their affiliation with one of the cancer-related pathways. Yellow nodes in the network highlight pathway elements linking two or more pathways to each other.

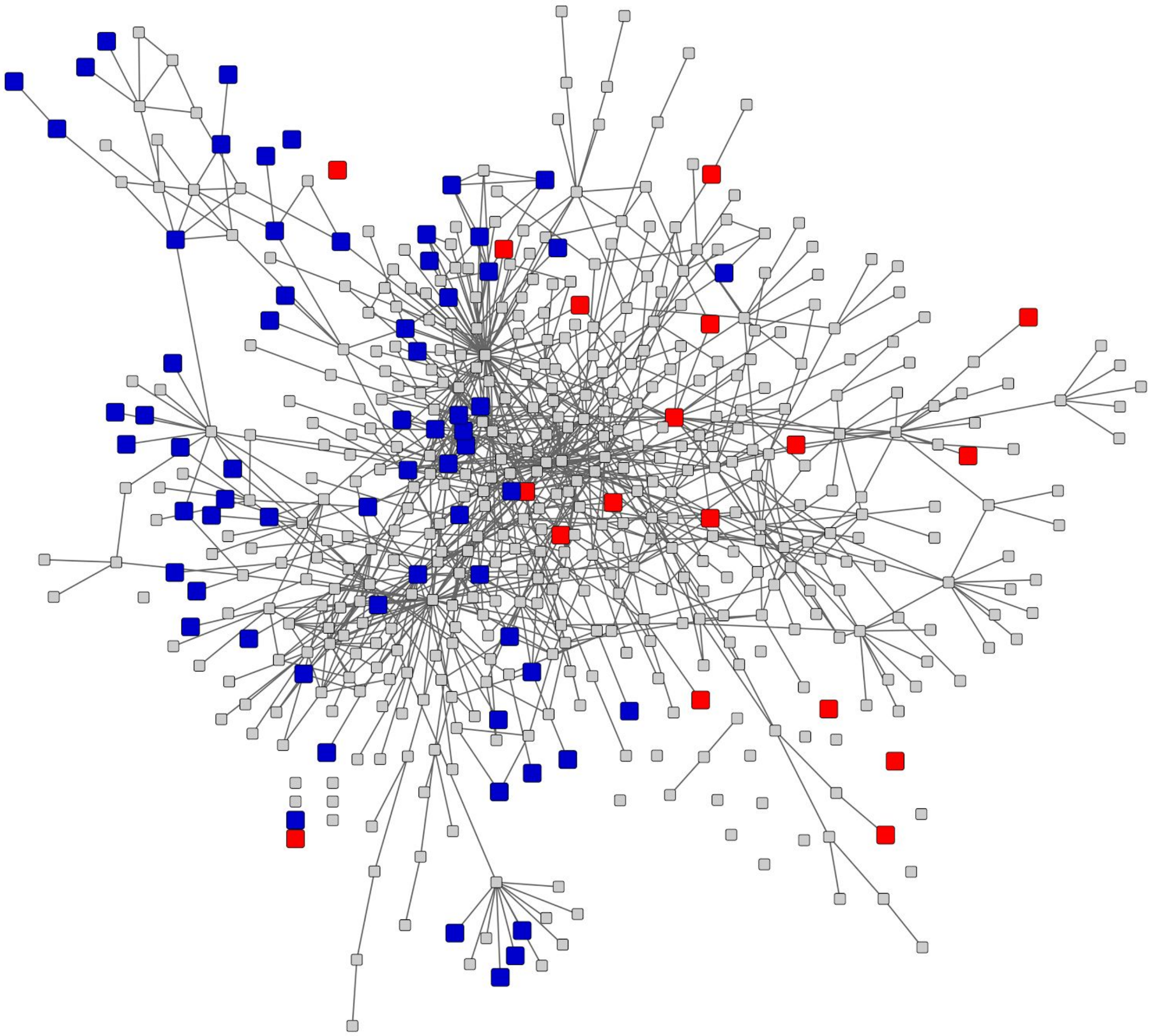

Figure S3B: Up- and down-regulated genes highlighted in cancer-related network

Pathway analysis revealed seven significantly altered cancer-related pathways in 1,25$(OH)_2D_3$-treated cancer cells (RB in Cancer, Gastric cancer network 1, Gastric cancer network 2, Integrated Pancreatic Cancer Pathway, Integrated Cancer Pathway, Integrated Breast Cancer Pathway, Signaling Pathways in Glioblastoma). The pathways were merged into one network using the WikiPathways and BridgeDb apps for Cytoscape. 18 genes in the network are up-regulated (red) and 66 genes are down-regulated (blue) in 1,25$(OH)_2D_3$-treated cancer cells.
