# Supplementary material for: Integrative network-based analysis of mRNA and microRNA expression in 1,25-dihydroxyvitamin D3-treated cancer cells
Source: Genes Nutr. 2015 Aug 15;10(5):35. doi: 10.1007/s12263-015-0484-0 (PMC4537452; doi:10.1007/s12263-015-0484-0)
Supplement: Supplementary file 4 — Supplementary material 4 (PDF 724 kb) [file 12263_2015_484_MOESM4_ESM.pdf]

Supplementary Material 4: Vitamin D-extended network

Article Title: Integrative network-based analysis of mRNA and microRNA expression in 1,25-dihydroxyvitamin D3-treated cancer cells

Journal name: Genes & Nutrition

Author Names: Martina Kutmon, Susan L Coort, Kim de Nooijer, Claire Lemmens, Chris T Evelo

Email address: martina.kutmon@maastrichtuniversity.nl

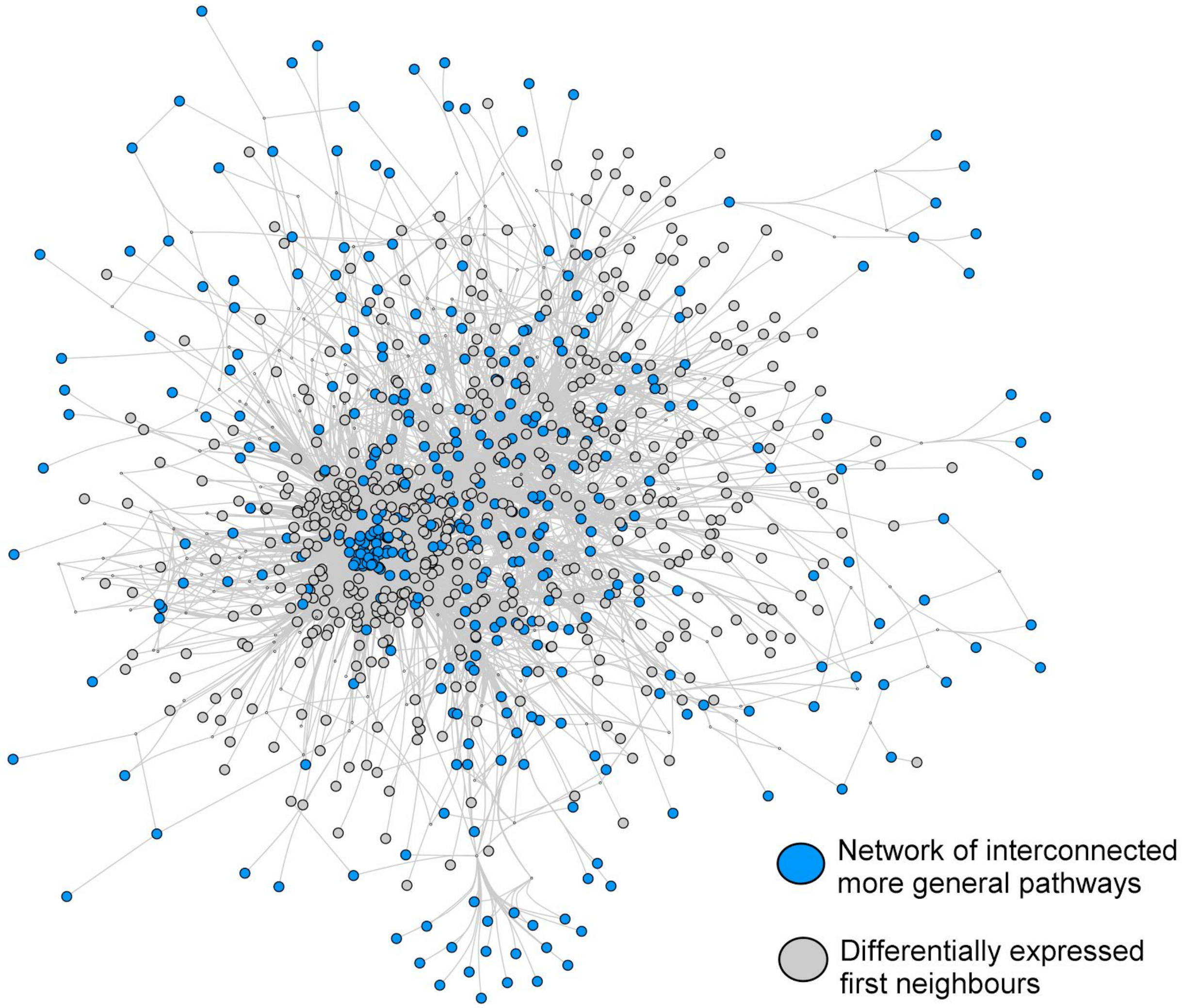

Figure S4A: Merged pathways were extended with first neighbours. The extended network consists of 1,013 nodes and 9,200 edges. 503 nodes (blue) from the selected more general (cell cycle related) pathways were extended with 510 nodes (gray) from STRING and ENCODE.

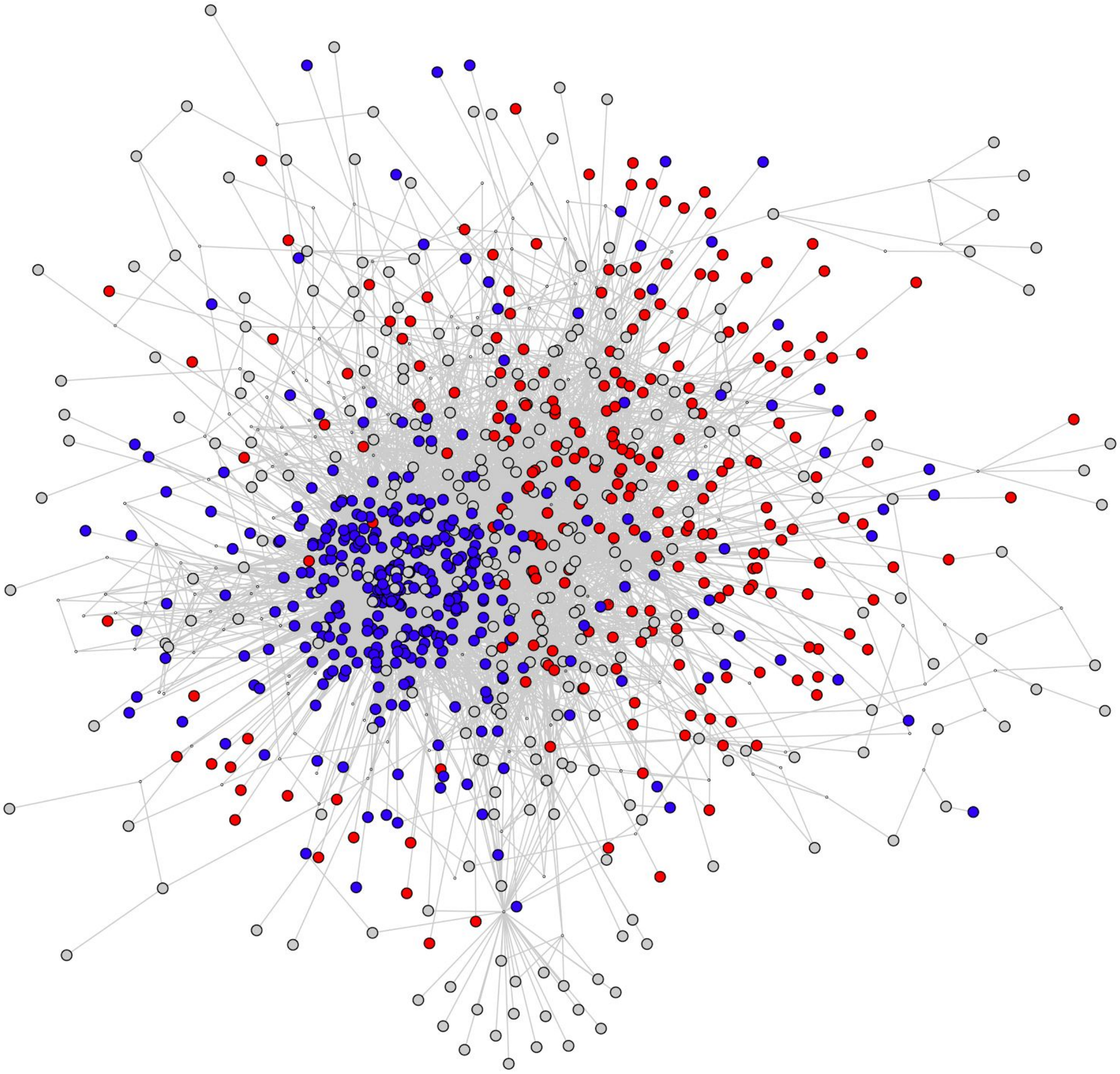

Figure S4B: Differentially expressed genes in the vitamin D-extended network. The extended network contains 238 up- and 345 down-regulated genes. 70% of all differentially expressed genes in 1,25(OH)$_2$D$_3$-treated cancer cells are now present in the network.
