# Supplementary material for: Integrative network-based analysis of mRNA and microRNA expression in 1,25-dihydroxyvitamin D3-treated cancer cells
Source: Genes Nutr. 2015 Aug 15;10(5):35. doi: 10.1007/s12263-015-0484-0 (PMC4537452; doi:10.1007/s12263-015-0484-0)
Supplement: Supplementary file 5 — Supplementary material 5 (PDF 207 kb) [file 12263_2015_484_MOESM5_ESM.pdf]

Supplementary Material 5: GO processes for active module

Article Title: Integrative network-based analysis of mRNA and microRNA expression in 1,25-dihydroxyvitamin D3-treated cancer cells
Journal name: Genes & Nutrition
Author Names: Martina Kutmon, Susan L Coort, Kim de Nooijer, Claire Lemmens, Chris T Evelo
Email address: martina.kutmon@maastrichtuniversity.nl

Function	Group Genes
**DNA catabolic process, exonucleolytic**	*BLM, DNA2, EXO1, LIG1, NEIL3, PCNA, RAD51C*
**DNA duplex unwinding**	*BLM, DNA2, KIF18A, KIF20B, LIG1, MCM2, MCM4, MCM7, MSH5, PCNA, PIF1, POLE2, POLQ, PRIM1, RAD51, RFC3, RFC4, TK1, TOP2A*
**DNA metabolic process**	*ASF1B, ASPM, BLM, BRCA1, BRCA2, CASC5, CCNB1, CCNE2, CDC25C, CDC45, CDC6, CDK1, CDT1, CENPA, CENPF, CENPH, CENPI, CENPK, CENPO, CENPW, CHEK2, CHTF18, DEPDC1B, DNA2, DSCC1, DTL, E2F7, E2F8, EME1, ERCC6L, ESCO2, EXO1, FANCA, FANCB, FANCD2, FANCG, FOXM1, GINS1, GINS3, GMNN, HELLS, HJURP, KIAA0101, LIG1, LIN9, MCM10, MCM2, MCM3, MCM4, MCM7, MDC1, MKI67, MND1, MSH5, NCAPD2, NCAPG, NEIL3, NUSAP1, OIP5, ORC1, ORC6, PCNA, PIF1, PLK4, POLE2, POLQ, PRIM1, PTTG1, RAD51, RAD51AP1, RAD51C, RAD54L, RFC3, RFC4, RMI2, RRM2, SMC2, TICRR, TK1, TONSL, TOP2A, TRIP13, USP1, ZNF367*
**DNA packaging**	*ASF1B, CASC5, CCNB1, CDK1, CENPA, CENPE, CENPH, CENPI, CENPK, CENPO, CENPW, DLGAP5, DSCC1, ESPL1, GSG2, HELLS, HJURP, KIF18A, KIFC1, MCM2, MCM7, NCAPD2, NCAPG, NDC80, NEK2, NUSAP1, OIP5, PLK1, PSRC1, RAD51C, SMC2, SPAG5, TOP2A, ZWINT*
**DNA repair**	*ATAD5, AURKB, BLM, BRCA1, BRCA2, C11orf82, CCNA2, CCNB1, CDC6, CDK1, CDT1, CHEK2, DEPDC1B, DNA2, DSCC1, DTL, E2F7, E2F8, ECT2, EME1, ERCC6L, ESCO2, EXO1, FANCA, FANCB, FANCD2, FANCG, FOXM1, GMNN, GTSE1, KIAA0101, LIG1, MCM7, MDC1, MND1, MSH5, NEIL3, NMU, PCNA, PIF1, PLK1, POLE2, POLQ, PRIM1, PTTG1, RAD51, RAD51AP1, RAD51C, RAD54L, RFC3, RFC4, SMC2, TICRR, TONSL, TOP2A, TRIP13, USP1, ZNF367*
**DNA replication**	*AURKB, BLM, BRCA1, BRCA2, C11orf82, CCNA2, CCNB1, CCNE2, CCNF, CDC25B, CDC25C, CDC45, CDC6, CDK1, CDT1, CENPF, CENPJ, CEP152, CHEK2, CHTF18, DEPDC1B, DNA2, DSCC1, DTL, E2F7, E2F8, ECT2, ESCO2, FANCD2, FANCG, FOXM1, GINS1, GINS3, GMNN, GTSE1, KIAA0101, LIG1, LIN9, MCM10, MCM2, MCM3, MCM4, MCM7, MDC1, NMU, ORC1, ORC6, PCNA, PIF1, PLK4, POLE2, POLQ, PRIM1, RAD51, RAD51AP1, RAD51C, RAD54L, RFC3, RFC4, RMI2, RRM2, TICRR, TK1, TONSL, TOP2A, TRIP13, USP1*
**DNA-dependent DNA replication**	*BLM, BRCA1, BRCA2, CCNB1, CCNE2, CDC25C, CDC45, CDC6, CDK1, CDKN3, CDT1, CENPF, CHEK2, CHTF18, DNA2, DSCC1, DTL, E2F4, E2F7, E2F8, EME1, ESCO2, EXO1, FBXO5, GINS1, GINS3, GMNN, GSG2, GTSE1, IQGAP3, KIAA0101, KIF18A, KIF20B, LIG1, LIN9, MCM10, MCM2, MCM3, MCM4, MCM7, MDC1, MND1, MSH5, NEIL3, ORC1, ORC6, PCNA, PIF1, PKMYT1, POLE2, POLQ, PRIM1, RAD51, RAD51AP1, RAD51C, RAD54L, RFC3, RFC4, RMI2, RRM2, SMC2, TICRR, TK1, TONSL, TOP2A, TRIP13*
**blastocyst development**	*BRCA2, GINS1, NEK2*
**cell cycle**	*ANLN, ASPM, AUNIP, AURKA, AURKB, BLM, BRCA1, BRCA2, BUB1, BUB1B, C11orf82, CASC5, CCNA2, CCNB1, CCNE2, CCNF, CDC25B, CDC25C, CDC45, CDC6, CDCA2, CDCA3, CDCA7, CDCA8, CDK1, CDKN3, CDT1, CENPA, CENPE, CENPF, CENPH, CENPI, CENPJ, CENPK, CENPL, CENPM, CENPO, CENPW, CEP152, CEP55, CHEK2, CHTF18, DEPDC1B, DIAPH3, DLGAP5, DNA2, DSCC1, DTL, E2F4,*

	E2F7, E2F8, ECT2, ERCC6L, ESCO2, ESPL1, EXO1, FAM83D, FANCA, FANCD2, FANCG, FBXO5, FOXM1, GINS1, GMNN, GPSM2, GSG2, GTSE1, HAUS8, HELLS, HJURP, INCENP, IQGAP3, KIAA0101, KIF11, KIF15, KIF18A, KIF18B, KIF20A, KIF20B, KIF23, KIF24, KIF2C, KIFC1, LIG1, LIN9, MCM10, MCM2, MCM3, MCM4, MCM7, MDC1, MELK, MKI67, MND1, MSH5, NCAPD2, NCAPG, NDC80, NEK2, NUF2, NUSAP1, OIP5, ORC1, ORC6, PBK, PCNA, PHF19, PIF1, PKMYT1, PLK1, PLK4, POLE2, PRC1, PRIM1, PSRC1, PTTG1, RACGAP1, RAD51, RAD51C, RAD54L, RBL1, RFC3, RFC4, RRM2, SGOL1, SGOL2, SKA1, SKA3, SMC2, SPAG5, SPC25, SUV39H1, TACC3, TICRR, TOP2A, TPX2, TRIP13, TTK, UBE2C, VRK1, WDHD1, ZNF367, ZWINT
**cell cycle checkpoint**	*AURKB, BLM, BRCA1, BRCA2, BUB1, BUB1B, CASC5, CCNA2, CCNB1, CCNE2, CCNF, CDC45, CDC6, CDK1, CDT1, CENPF, CHEK2, DEPDC1B, DLGAP5, DNA2, DTL, E2F7, E2F8, ESPL1, FANCG, FBXO5, FOXM1, GSG2, GTSE1, MDC1, MKI67, PLK1, RAD51C, TICRR, TTK, UBE2C, ZNF367, ZWINT*
**cell cycle phase**	*ASPM, AURKA, AURKB, BLM, CCNB1, CDCA8, CENPA, CENPE, CENPF, DLGAP5, ESPL1, GPSM2, GTSE1, KIF18A, KIF20B, MSH5, NDC80, NUSAP1, PSRC1, SKA1, SKA3, TACC3, TPX2*
**cellular response to DNA damage stimulus**	*ASF1B, ATAD5, AURKB, BLM, BRCA1, BRCA2, C11orf82, CASC5, CCNA2, CCNB1, CCNE2, CDC25C, CDC45, CDC6, CDK1, CDT1, CENPA, CENPF, CENPH, CENPI, CENPK, CENPO, CENPW, CHEK2, CHTF18, DEPDC1B, DNA2, DSCC1, DTL, E2F7, E2F8, ECT2, EME1, ERCC6L, ESCO2, EXO1, FANCA, FANCB, FANCD2, FANCG, FOXM1, GINS1, GINS3, GMNN, GTSE1, HELLS, HJURP, KIAA0101, LIG1, LIN9, MCM10, MCM2, MCM3, MCM4, MCM7, MDC1, MKI67, MND1, MSH5, NCAPD2, NCAPG, NEIL3, NMU, NUSAP1, OIP5, ORC1, ORC6, PCNA, PIF1, PLK1, POLE2, POLQ, PRIM1, PTTG1, RAD51, RAD51AP1, RAD51C, RAD54L, RFC3, RFC4, RMI2, RRM2, SMC2, TICRR, TK1, TONSL, TOP2A, TRIP13, USP1, ZNF367*
**chromosome organization**	*ANLN, ASF1B, AURKB, BLM, BRCA1, BRCA2, BUB1, CASC5, CCNB1, CDCA8, CDK1, CENPA, CENPE, CENPF, CENPH, CENPI, CENPK, CENPO, CENPW, CHEK2, DEPDC1B, DLGAP5, DNA2, DSCC1, E2F4, ESPL1, FANCD2, FBXO5, GSG2, HAUS8, HELLS, HIRIP3, HJURP, KIF11, KIF18A, KIF23, KIF24, KIFC1, LIG1, MCM2, MCM4, MCM7, MSH5, NCAPD2, NCAPG, NDC80, NEK2, NUSAP1, OIP5, PCNA, PHF19, PIF1, PLK1, POLE2, PRIM1, PSRC1, PTTG1, RACGAP1, RAD51, RAD51C, RAD54L, RBL1, RFC3, RFC4, SMC2, SPAG5, SUV39H1, TOP2A, TRIP13, WDHD1, ZWINT*
**chromosome segregation**	*ASF1B, AURKB, BLM, BRCA1, BRCA2, BUB1, CASC5, CCNB1, CDC6, CDCA8, CDK1, CENPA, CENPE, CENPF, CENPH, CENPI, CENPK, CENPO, CENPW, DEPDC1B, DLGAP5, DNA2, DSCC1, ECT2, ESPL1, FANCD2, GSG2, HELLS, HIRIP3, HJURP, INCENP, KIF18A, KIF2C, KIFC1, LIG1, MCM2, MCM4, MCM7, MSH5, NCAPD2, NCAPG, NDC80, NEK2, NUF2, NUSAP1, OIP5, PCNA, PHF19, PIF1, PLK1, POLE2, PRIM1, PSRC1, PTTG1, RACGAP1, RAD51, RAD51C, RAD54L, RBL1, RFC3, RFC4, SGOL1, SGOL2, SKA1, SKA3, SMC2, SPAG5, SPC25, SUV39H1, TOP2A, TRIP13, WDHD1, ZWINT*
**cytoskeleton organization**	*ANLN, ASPM, AUNIP, AURKA, AURKB, BRCA1, BRCA2, BUB1, BUB1B, CASC5, CCNB1, CCNF, CDC25B, CDC25C, CDC6, CDK1, CENPA, CENPF, CENPJ, CEP152, CHEK2, DIAPH3, DLGAP5, ECT2, ESPL1, FBXO5, GPSM2, HAUS8, KIAA0101, KIF11, KIF18A, KIF18B, KIF20A, KIF20B, KIF23, KIF24, KIF2C, NCAPG, NDC80, NEK2, NUSAP1, PHF19, PIF1, PKMYT1, PLK1, PLK4, PRC1, PSRC1, RACGAP1, SGOL1, SKA1, SKA3, SPAG5, SPC25, TACC3, TONSL, TPX2, TTK, UBE2C, WDHD1, ZWINT*
**meiosis I**	*BRCA2, CDC25B, ESPL1, FANCA, FANCD2, FBXO5, LIG1, MSH5, RAD51, RAD51C, SGOL1, SMC2, TOP2A, TRIP13*
**meiotic nuclear division**	*ASPM, AURKA, BRCA2, C11orf82, CASC5, CCNB1, CDC25B, CDK1, CHEK2, DLGAP5, ESPL1, EXO1, FANCA, FANCD2, FANCG, FBXO5, FOXM1, MKI67, MND1, MSH5, NEK2, NUSAP1, PLK1, PSRC1, RAD51, RAD51C, RAD54L, SGOL1, SGOL2, SMC2, TK1, TOP2A, TRIP13, VRK1*
**microtubule bundle formation**	*KIF20A, PLK1, PSRC1*
**microtubule cytoskeleton organization**	*ANLN, ASPM, AUNIP, AURKA, AURKB, BLM, BRCA1, BRCA2, BUB1, BUB1B, C11orf82, CASC5, CCNA2, CCNB1, CCNE2, CCNF, CDC25B, CDC25C, CDC45, CDC6, CDK1, CDKN3, CDT1, CENPA, CENPF, CENPJ, CEP152, CEP55, CHEK2, DEPDC1B, DIAPH3, DLGAP5, DNA2, DTL, E2F4, E2F7, E2F8, ECT2, ESPL1, FANCG, FBXO5, FOXM1, GMNN, GPSM2, GSG2, GTSE1, HAUS8, INCENP, IQGAP3, KIAA0101,*

	KIF11, KIF18A, KIF18B, KIF20A, KIF20B, KIF23, KIF24, KIF2C, LIN9, MCM10, MCM2, MCM3, MCM4, MCM7, MDC1, MELK, NCAPG, NDC80, NEK2, NUSAP1, ORC1, ORC6, PCNA, PHF19, PIF1, PKMYT1, PLK1, PLK4, POLE2, PRC1, PRIM1, PSRC1, PTTG1, RACGAP1, RAD51C, RBL1, RRM2, SGOL1, SKA1, SKA3, SPAG5, SPC25, TACC3, TICRR, TK1, TPX2, TTK, UBE2C, VRK1, WDHD1, ZNF367, ZWINT
**microtubule polymerization**	*CENPJ, FBXO5, PSRC1*
**microtubule-based process**	*ANLN, AUNIP, AURKA, AURKB, BRCA1, BRCA2, BUB1B, CASC5, CCNB1, CCNF, CDK1, CENPA, CENPE, CENPJ, CEP152, CHEK2, DIAPH3, DLGAP5, ECT2, ESPL1, FBXO5, GPSM2, GTSE1, HAUS8, KIAA0101, KIF11, KIF14, KIF15, KIF18A, KIF18B, KIF20A, KIF20B, KIF23, KIF24, KIF2C, KIF4A, KIFC1, NCAPG, NDC80, NEK2, NUSAP1, PLK1, PLK4, PRC1, PSRC1, RACGAP1, SGOL1, SKA1, SKA3, SPAG5, SPC25, TACC3, TPX2, TTK, UBE2C, ZWINT*
**mismatch repair**	*EXO1, MSH5, PCNA*
**mitotic cell cycle**	*ANLN, ASF1B, ASPM, AUNIP, AURKA, AURKB, BLM, BRCA1, BRCA2, BUB1, BUB1B, C11orf82, CASC5, CCNA2, CCNB1, CCNE2, CCNF, CDC25B, CDC25C, CDC45, CDC6, CDCA2, CDCA3, CDCA7, CDCA8, CDK1, CDKN3, CDT1, CENPA, CENPE, CENPF, CENPH, CENPI, CENPJ, CENPK, CENPL, CENPM, CENPO, CENPW, CEP152, CEP55, CHEK2, DEPDC1B, DIAPH3, DLGAP5, DNA2, DSCC1, DTL, E2F4, E2F7, E2F8, ECT2, ERCC6L, ESCO2, ESPL1, EXO1, FAM83D, FANCA, FANCD2, FANCG, FBXO5, FOXM1, GINS1, GMNN, GPSM2, GSG2, GTSE1, HAUS8, HELLS, HIRIP3, HJURP, INCENP, IQGAP3, KIAA0101, KIF11, KIF14, KIF15, KIF18A, KIF18B, KIF20A, KIF20B, KIF23, KIF24, KIF2C, KIF4A, KIFC1, LIG1, LIN9, MCM10, MCM2, MCM3, MCM4, MCM7, MDC1, MELK, MKI67, MND1, MSH5, NCAPD2, NCAPG, NDC80, NEK2, NUF2, NUSAP1, OIP5, ORC1, ORC6, PBK, PCNA, PHF19, PIF1, PKMYT1, PLK1, PLK4, POLE2, PRC1, PRIM1, PSRC1, PTTG1, RACGAP1, RAD51, RAD51C, RAD54L, RBL1, RFC3, RFC4, RRM2, SGOL1, SGOL2, SKA1, SKA3, SMC2, SPAG5, SPC25, SUV39H1, TACC3, TICRR, TK1, TOP2A, TPX2, TRIP13, TTK, UBE2C, VRK1, WDHD1, ZNF367, ZWINT*
**mitotic cell cycle phase transition**	*AURKA, BLM, BRCA2, BUB1, BUB1B, CCNA2, CCNB1, CCNE2, CDC25B, CDC25C, CDC45, CDC6, CDK1, CDKN3, CDT1, CENPF, CENPJ, CEP152, CHEK2, DLGAP5, DNA2, E2F4, E2F7, E2F8, ESPL1, FBXO5, FOXM1, GINS1, GSG2, GTSE1, IQGAP3, LIG1, LIN9, MCM10, MCM2, MCM3, MCM4, MCM7, MDC1, MELK, NEK2, ORC1, ORC6, PCNA, PKMYT1, PLK1, PLK4, POLE2, POLQ, PRIM1, RAD51, RAD51C, RFC3, RFC4, RRM2, TICRR, TK1, TONSL, TOP2A, TTK, UBE2C, ZWINT*
**mitotic recombination**	*BLM, DNA2, LIG1, MSH5, PCNA, POLE2, PRIM1, RAD51, RAD51C, RFC3, RFC4, TOP2A, TRIP13*
**mitotic sister chromatid segregation**	*ASPM, AURKA, BRCA2, BUB1B, CCNB1, CDC25B, CDK1, CENPA, CENPE, CENPF, DLGAP5, DSCC1, ESPL1, GPSM2, GSG2, KIF18A, KIFC1, NCAPD2, NCAPG, NDC80, NEK2, NUSAP1, PLK1, PSRC1, RAD51C, SMC2, SPAG5, TACC3, TOP2A, ZWINT*
**mitotic spindle organization**	*AURKA, AURKB, BRCA1, BUB1, BUB1B, CASC5, CCNB1, CCNF, CDK1, CENPE, CENPF, CENPJ, CHEK2, ECT2, ESPL1, FBXO5, GSG2, KIF11, KIF18A, KIF18B, KIF23, KIF24, KIF2C, NCAPG, NDC80, NEK2, PHF19, PIF1, PLK1, PLK4, PRC1, PSRC1, PTTG1, RACGAP1, SKA1, SKA3, SPAG5, SPC25, TACC3, TPX2, TTK, UBE2C*
**negative regulation of organelle organization**	*AURKA, AURKB, BRCA1, BUB1, BUB1B, CCNB1, CCNF, CENPF, ESPL1, FBXO5, NCAPG, PHF19, PIF1, PLK1, TTK, UBE2C, WDHD1*
**neural precursor cell proliferation**	*ASPM, MELK, RACGAP1, SHCBP1, TACC3*
**peptidyl-tyrosine dephosphorylation**	*CDC25B, CDC25C, CDKN3, ZNF367*
**phosphatidylinositol-mediated signaling**	*AURKA, BUB1B, NDC80, PCNA, RFC4, SPAG5, TOP2A, UBE2C, ZWINT*
**regulation of G2/M transition of mitotic cell cycle**	*CCNA2, CDK1, CENPF, CHEK2, GTSE1, MDC1, MKI67, RAD51C, TICRR*
**regulation of cell cycle process**	*ANLN, ASPM, AUNIP, AURKA, AURKB, BLM, BRCA1, BRCA2, BUB1, BUB1B, CASC5, CCNA2, CCNB1, CCNF, CDC25B, CDC25C, CDC6, CDK1, CDT1, CENPA, CENPE, CENPF, CENPH, CENPJ, CENPW, CEP55, CHEK2, DLGAP5, E2F4, E2F7, E2F8, ECT2, ESPL1, FBXO5,*

	FOXM1, GPSM2, GSG2, GTSE1, HAUS8, INCENP, KIF11, KIF14, KIF15, KIF18A, KIF18B, KIF20A, KIF20B, KIF23, KIF24, KIF2C, KIF4A, KIFC1, MDC1, NDC80, NEK2, NUSAP1, PKMYT1, PLK1, PLK4, PRC1, PSRC1, RACGAP1, RAD51C, SPAG5, SPC25, TACC3, TICRR, TPX2, TTK, UBE2C, ZWINT
**regulation of cyclin-dependent protein serine/threonine kinase activity**	*BLM, CCNA2, CCNB1, CCNE2, CDC25C, CDC6, CDKN3, MCM2, PKMYT1, PLK1, PSRC1*
**regulation of microtubule cytoskeleton organization**	*AURKA, AURKB, BRCA1, BRCA2, CCNB1, CCNF, CDK1, CENPJ, CEP152, ECT2, FOXM1, HAUS8, KIAA0101, KIF11, NCAPG, NEK2, PLK1, PLK4, PSRC1, RACGAP1, SGOL1, SKA1, SKA3, SPAG5, TACC3, TPX2*
**response to ionizing radiation**	*BLM, BRCA1, BRCA2, C11orf82, CDK1, CHEK2, DEPDC1B, ECT2, FANCD2, FOXM1, RAD51, RAD51AP1, RAD54L, TICRR*
**response to radiation**	*AURKB, BLM, BRCA1, BRCA2, C11orf82, CHEK2, DEPDC1B, DTL, ECT2, FANCD2, FANCG, KIAA0101, NMU, PCNA, POLQ, RAD51, RAD51AP1, RAD54L, RFC3, TICRR, USP1*
**single-organism organelle organization**	*ANLN, ASPM, AUNIP, AURKA, AURKB, BLM, BRCA1, BRCA2, BUB1, BUB1B, CASC5, CCNA2, CCNB1, CCNF, CDC25B, CDC25C, CDC6, CDCA2, CDCA3, CDCA8, CDK1, CENPA, CENPE, CENPF, CENPJ, CENPW, CEP152, CEP55, CHEK2, DEPDC1B, DIAPH3, DLGAP5, DNA2, DSCC1, ECT2, ERCC6L, ESPL1, FAM83D, FANCD2, FBXO5, GPSM2, GSG2, GTSE1, HAUS8, HELLS, INCENP, KIAA0101, KIF11, KIF14, KIF15, KIF18A, KIF18B, KIF20A, KIF20B, KIF23, KIF24, KIF2C, KIF4A, KIFC1, LIG1, MSH5, NCAPD2, NCAPG, NDC80, NEK2, NUF2, NUSAP1, OIP5, PBK, PCNA, PHF19, PIF1, PKMYT1, PLK1, PLK4, POLE2, PRC1, PRIM1, PSRC1, PTTG1, RACGAP1, RAD51, RAD51C, RFC3, RFC4, SGOL1, SKA1, SKA3, SMC2, SPAG5, SPC25, TACC3, TOP2A, TPX2, TRIP13, TTK, UBE2C, VRK1, WDHD1, ZWINT*
**sister chromatid cohesion**	*BUB1, DSCC1, ESPL1, GSG2, RAD51C*
**sister chromatid segregation**	*ASPM, AURKB, BRCA1, BUB1, CASC5, CCNB1, CDC6, CDK1, CENPA, CENPE, CENPF, CENPW, DLGAP5, DSCC1, ECT2, ESPL1, GPSM2, GSG2, HJURP, INCENP, KIF18A, KIF2C, KIFC1, MSH5, NCAPD2, NCAPG, NDC80, NEK2, NUF2, NUSAP1, PLK1, PSRC1, PTTG1, RACGAP1, RAD51C, SGOL1, SGOL2, SKA1, SKA3, SMC2, SPAG5, SPC25, TACC3, TOP2A, ZWINT*
**spindle assembly involved in mitosis**	*ANLN, AURKB, CASC5, CENPA, CENPE, CENPF, CENPH, CENPW, CHEK2, E2F4, FBXO5, HAUS8, KIF11, KIF23, KIF24, NEK2, RACGAP1, SMC2*
**spindle checkpoint**	*AURKA, AURKB, BUB1, BUB1B, CASC5, CCNB1, CDK1, CENPA, CENPF, ESPL1, FBXO5, GPSM2, GSG2, NDC80, NUSAP1, PLK1, PTTG1, TTK, UBE2C, ZNF367*
**spindle organization**	*AUNIP, AURKA, AURKB, BUB1B, CASC5, CCNB1, CHEK2, ECT2, ESPL1, FBXO5, HAUS8, KIF11, KIF23, NDC80, NEK2, PRC1, PSRC1, RACGAP1, SGOL1, SPAG5, SPC25, TACC3, TPX2, TTK, UBE2C, ZWINT*
